# Single-cell transcriptomics reveals the role of Macrophage-Naïve CD4 + T cell interaction in the immunosuppressive microenvironment of primary liver carcinoma

**DOI:** 10.1186/s12967-022-03675-2

**Published:** 2022-10-11

**Authors:** Zhuomao Mo, Daiyuan Liu, Yihan Chen, Jin Luo, Wenjing Li, Jiahui Liu, Ling Yu, Bijun Huang, Shijun Zhang

**Affiliations:** 1 Department of Traditional Chinese Medicine, The First Affiliated Hospital, Sun Yat-Sen University, 58 Zhongshan Road II, Guangzhou, 510080 Guangdong province China; 2 Center for Stem Cell and Regenerative Medicine, Zhejiang University School of Medicine, Hangzhou, 310058 Zhejiang province China; 3 Department of Biochemistry, Zhongshan School of Medicine, Sun Yat-Sen University, Guangzhou, 510080 Guangdong province China; 4 Key Laboratory for Stem Cells and Tissue Engineering, Ministry of Education, Sun Yat-Sen University, Guangzhou, 510080 Guangdong province China; 5 Department of Histology and Embryology, Zhongshan School of Medicine, Sun Yat-Sen University, Guangzhou, 510080 Guangdong province China; 6 Guangdong Province Hospital of Chinese Medicine, AMI Key Laboratory of Chinese Medicine in Guangzhou, Guangzhou, 510120 Guangdong province China; 7 Department of Experimental Research, State Key Laboratory of Oncology in South China, Collaborative Innovation Center for Cancer Medicine, Sun Yat-Sen University Cancer Center, Guangzhou, 510060 Guangdong province China

**Keywords:** Single cell transcriptomic, Macrophage, Naïve CD4 + T cell, Tumor microenvironment, Immunosuppression

## Abstract

**Background:**

Liver carcinoma generally presents as an immunosuppressive microenvironment that promotes tumor evasion. The intercellular crosstalk of immune cells significantly influences the construction of an immunosuppressive microenvironment. This study aimed to investigate the important interactions between immune cells and their targeting drugs in liver carcinoma, by using single-cell and bulk transcriptomic data.

**Methods:**

Single-cell and bulk transcriptomic data were retrieved from Gene Expression Omnibus (GSE159977, GSE136103, and GSE125449) and The Cancer Genome Atlas (TGCA-LIHC), respectively. Quality control, dimension reduction, clustering, and annotation were performed according to the Scanpy workflow based on Python. Cell–cell interactions were explored using the CellPhone database and CellChat. Trajectory analysis was executed using a partition-based graph abstraction method. The transcriptomic factors (TFs) were predicted using single-cell regulatory network inference and clustering (SCENIC). The target genes from TFs were used to establish a related score based on the TCGA cohort; this score was subsequently validated by survival, gene set enrichment, and immune cell infiltration analyses. Drug prediction was performed based on the Cancer Therapeutics Response Portal and PRISM Repurposing datasets.

**Results:**

Thirty-one patients at four different states, including health, hepatitis, cirrhosis, and cancer, were enrolled in this study. After dimension reduction and clustering, twenty-two clusters were identified. Cell–cell interaction analyses indicated that macrophage-naive CD4 + T cell interaction significantly affect cancerous state. In brief, macrophages interact with naive CD4 + T cells via different pathways in different states. The results of SCENIC indicated that macrophages present in cancer cells were similar to those present during cirrhosis. A macrophage-naive CD4 + T cell (MNT) score was generated by the SCENIC-derived target genes. Based on the MNT score, five relevant drugs (inhibitor of polo-like kinase 1, inhibitor of kinesin family member 11, dabrafenib, ispinesib, and epothilone-b) were predicted.

**Conclusions:**

This study reveals the crucial role of macrophage-naive CD4 + T cell interaction in the immunosuppressive microenvironment of liver carcinoma. Tumor-associated macrophages may be derived from cirrhosis and can initiate liver carcinoma. Predictive drugs that target the macrophage-naive CD4 + T cell interaction may help to improve the immunosuppressive microenvironment and prevent immune evasion. The relevant mechanisms need to be further validated in experiments and cohort studies.

**Graphical Abstract:**

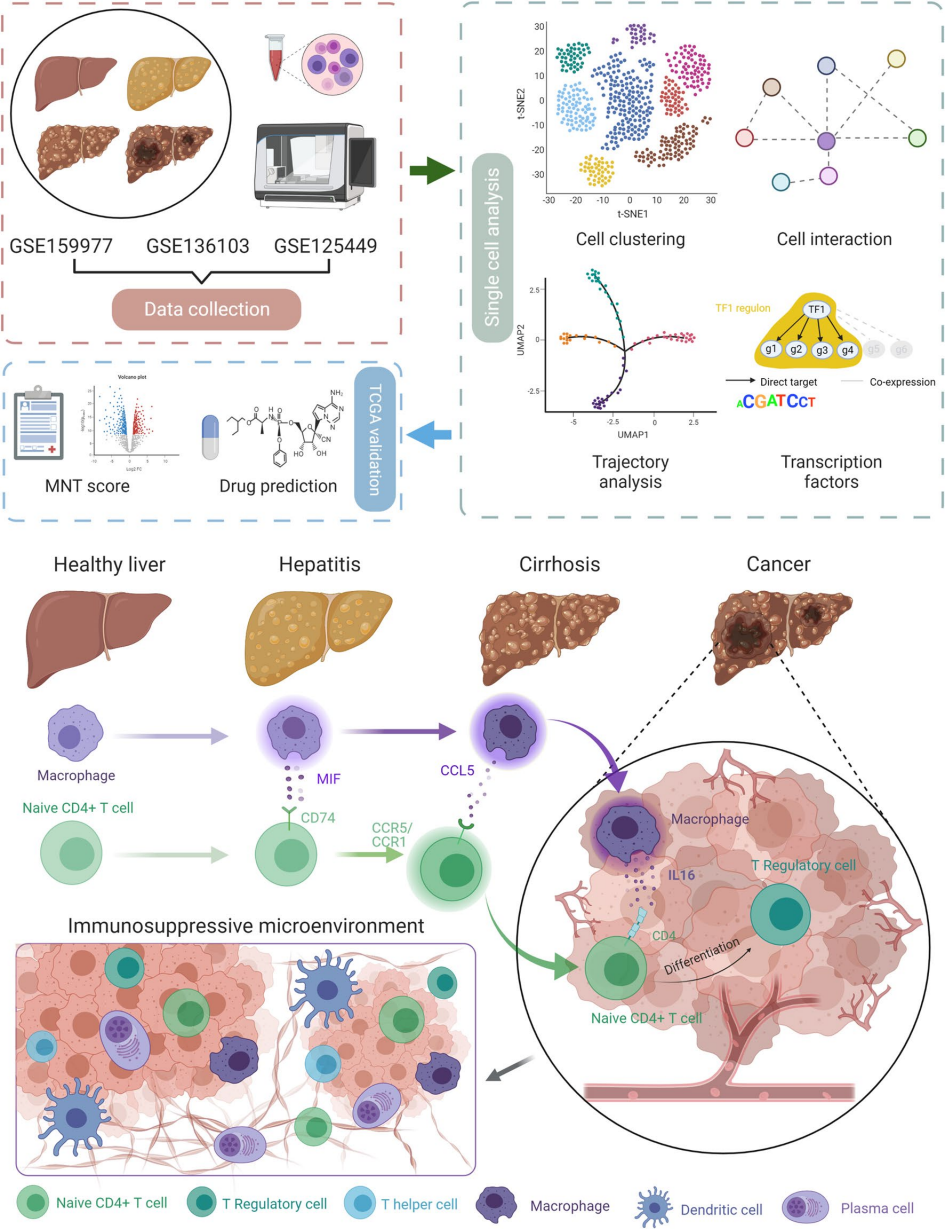

**Supplementary Information:**

The online version contains supplementary material available at 10.1186/s12967-022-03675-2.

## Introduction

Liver cancer remains the fourth most common cause of tumor-mediated death and ranks sixth in terms of incidence worldwide [1]. As the first-line treatment for liver cancer, sorafenib prolongs survival modestly compared with placebo, conferring very limited survival benefits [2]. Several immune checkpoint inhibitors have been approved for treating various cancers. Nivolumab, pembrolizumab, and atezolizumab, immune checkpoint inhibitors of the PDL1–PD1 pathway, have shown impressive responses against tumors and have a manageable safety profile in liver cancer [3–5]. However, it has been reported that patients with nonalcoholic steatohepatitis (NASH)-mediated liver cancer may not benefit from anti-PD1 treatment; besides, NASH-related aberrant T-cell activation is a potential cause [6]. The immune context should be recognized as an important determinant of immunotherapy efficacy. To identify patients who would benefit most from immunotherapeutic interventions, it is important to elucidate the composition and properties of cells in the tumor microenvironment (TME).

Most patients with liver cancer have cirrhosis or chronic hepatitis [7]. A complex balance exists between immunity and tolerance in healthy livers, and with progression from hepatitis to cirrhosis and liver cancer, the liver immune microenvironment gets changed. With functions of maintaining tolerance, immune suppression, and oncogenesis, immune cells such as macrophages, T regulatory (Treg) cells, and myeloid-derived suppressor cells (MDSCs) are constantly activated. Cells that contribute to protective antimicrobial or antitumor immunity, such as natural killer (NK) cells and T effector (Teff) cells, are mostly inhibited [8]. Multiple suppressive cells and molecules function in the TME, thus impairing antitumor responses [9]. Nevertheless, how these cells and molecules change in the dynamic and highly complex microenvironment of a healthy liver that converts to a cancerous liver, remains poorly understood.

Here, we integrated 31 cases of single-cell transcriptomic data from healthy patients and those with hepatitis, cirrhosis, and cancer. Interestingly, we found that macrophage-naïve CD4 + T cell interaction significantly influenced TME, which presented various communication pathways in different states. On the one hand, this interaction became active in cirrhosis and cancer; on the other hand, the differentiation of Treg cells from naïve CD4 + T cell occurred first in cirrhosis and then became more obvious in cancer. Consequently, we hypothesized that naïve CD4 + T cell induced by Macrophage differentiated into Treg in cirrhosis and they finally contributed to immunosuppressive TME. Furthermore, we generated a score to quantify the macrophage-naïve CD4 + T cell interaction and predicted the drugs targeting this interaction. Our results enable to shed light on the key interactions and sources of immunosuppressive TME in the liver, potentially assisting to prevent tumor evasion, and further guide the development of rational immunotherapy for primary liver carcinoma.

## Materials and methods

### Data collection

Single-cell transcriptomic data were retrieved from Gene Expression Omnibus (GEO; https://www.ncbi.nlm.nih.gov/geo/) database. Three datasets were employed in our research: GSE159977 [6], GSE136103 [10], and GSE125449 [11]. We selected the cases among these three datasets, finally settling on 31 cases (hepatitis = 3, cirrhosis = 5, health = 5, and cancer = 18) for this study. Besides, bulk transcriptomic data were retrieved from The Cancer Genome Atlas (TCGA-LIHC; https://portal.gdc.cancer.gov/), GEO databases (GSE25097; GSE33814; GSE54236) and ICGC database (LIRI cohort; https://dcc.icgc.org/). After data collection, we summarized and presented the workflow of this study (Additional file 1: Fig. S1). Simultaneously, we collected some available clinical features of employed datasets and samples from single-cell transcriptomic data in Additional file 1: Table S1.

### Single-cell RNA-seq data processing

We downloaded the three files (barcodes, features, and matrix) for each case and employed them to generate the object of Scanpy [12]. Considering the immune cells employed in one dataset (GSE159977), we only extracted the immune cells for further analyses. Genes expressed in fewer than three cells in a sample were excluded, as were cells that expressed fewer than 200 genes. Further quality control was performed on cells based on the number of genes expressed in the count matrix and the percentage of mitochondrial gene counts. Cells with > 2500 genes were filtered, as well as cells with > 5% of mitochondrial gene count (Additional file 1: Fig. S2A–E). We then applied the library-size correction method to normalize the data matrix using the “scanpy.pp.normalize_total” function in Scanpy. A logarithmized normalized data matrix was employed for downstream analysis.

### Dimension reduction and unsupervised clustering

Dimension reduction and unsupervised clustering were performed based on the workflow in Scanpy. We selected highly variable genes for downstream analysis by using the “scanpy.pp.highly_variable_genes” function with parameter “highly_variable_nbatches ≥ 4”. A total of 2303 highly variable genes were identified. To investigate the effect of the cell cycle, we calculated the “S_score” and “G2M_score” by using “sc.tl.score_genes_cell_cycle”. Then, the effects of total counts per cell, the percentage of mitochondrial genes expressed, the “S_score” and “G2M_score” were regressed out using the “scanpy.pp.regress_out” function. We also scaled each gene to unit variance using “scanpy.pp.scale” with parameter “max_value = 10”. After data preprocessing, we reduced the dimensionality of the data by performing a principal component analysis (PCA). A PCA matrix was calculated to reveal the main axes of variation and denoise the data through the “scanpy.tl.pca” function with parameter “svd_solver = ‘arpack’, n_pcs = 11” (Additional file 1: Fig. S2F). To remove the batch effects from different datasets, a BBKNN (Batch Balanced K Nearest Neighbors) algorithm with parameter “n_pcs = 35, metric = ‘euclidean” was performed. BBKNN is a fast and lightweight batch alignment method, which is written in Python and compatible with Scanpy, and its output can be immediately used for dimensionality reduction, clustering and pseudotime inference [13]. Previous study [14] has compared the different algorithms for batch correction and found that BBKNN showed the good performance in high number of batches and RAM usage. Furthermore, the dimensionality of merged datasets was reduced using uniform manifold approximation and projection (UMAP) implemented by the “scanpy.tl.umap” function. Then, to cluster the neighborhood graph of the cells, we employed the Leiden graph-clustering method. The marker genes of each cluster were identified using the “scanpy.tl.rank_genes_groups” function.

### Cell cluster annotation

We employed the marker genes that were validated experimentally and calculated from Scanpy to annotate cell clusters. Genes that were highly and specifically expressed in the cell cluster were considered as marker genes. CellMarker (http://biocc.hrbmu.edu.cn/CellMarker/) [15] and Panglao DB (https://panglaodb.se/) [16] websites were used to identify the cell types representing the marker genes.

### Cell–cell interaction analyses

We used CellPhone database [17] and CellChat [18] to infer cell–cell interactions between immune cell subsets. The potential interaction strength was predicted based on the expression of immune-associated receptors and ligands. The enriched ligand-receptor interactions were calculated based on a permutation test. *p* < 0.01 were considered significant. To further complement the cell–cell interaction analysis using CellPhoneDB, we employed the CellChat package for analysis. The Cellchat package includes a comprehensive signaling molecule interaction database that considers the known structural composition of receptor-ligand interactions, such as multimeric receptor-ligand complexes, soluble agonists and antagonists, as well as stimulatory and inhibitory membrane-bound coreceptors [18]. The inference of cell–cell interactions includes identification of differentially expressed signaling genes, calculation of ensemble average expression and intercellular communication probability, and identification of statistically significant intercellular communications. We divided the single-cell transcriptomic data into four parts according to the states (health, hepatitis, cirrhosis, and cancer) and explored the differences in ligand and receptor interactions and signaling pathways, across these four states.

### Gene regulatory network

To identify cell type-specific gene regulatory networks, single-cell regulatory network inference and clustering (SCENIC) analysis were performed. Genes that were expressed in 3% of samples and cells that expressed > 0 unique molecular identifier (UMI) and normalized by log_2_(filtered expr + 1) were filtered according to the SCENIC workflow [19]. Genes present in RcisTarget’s human feather databases (hg19-500 bp-upstream-7species.mc9nr.feather and hg19-tss-centered-10 kb-7species.mc9nr.feather) were utilized. These genes are predicted in a region of 500 to 10,000 base pairs, upstream of the hg19 human reference genome. The SCENIC workflow consisted of three steps: (1) identification of co-expression modules between transcription factor (TF) and potential target genes; (2) inference of direct target genes based on those potential targets for which the motif of the corresponding TF is significantly enriched (The regulon is defined as a TF and its direct target genes); (3) calculation of the regulon activity score in every single cell using the area under the recovery curve. To further quantify the cell-type specificity of a regulon, we adapted an entropy-based strategy that was previously used for gene expression data analysis, to generate a regulon-specific score (RSS) [20]. The algorithm was comprehensively described in a previous study [21].

### Trajectory analysis

To characterize the developmental trajectory between naïve CD4 + T cells and Treg cells, trajectory analysis was performed by partition-based graph abstraction (PAGA) [22] in this research. We employed the PAGA method of Scanpy to assess the most likely trajectories between naïve CD4 + T and Treg cells. The computations were performed using the default parameters.

### TCGA data analyses

#### Score generation and validation

To further validate the role of macrophage-naïve CD4 + T cell interaction in bulk transcriptomic data, we established a score based on the target genes of top regulons from these two cell types. First, differential expression analysis was performed to select the target genes. Genes with both *p* < 0.05, and |log_2_fold change|> 1 were considered significantly differentially expressed. Subsequently, we performed univariate Cox regression analysis to further explore the prognostic genes. Genes in the univariate Cox analysis were eligible for further selection if they exhibited a *p* < 0.01. The least absolute shrinkage and selection operator (LASSO) regression analysis was performed to establish the score. Here, a lasso penalty was used to account for shrinkage and variable selection. The optimal value of the lambda penalty parameter was defined by performing 10 cross-validations. The formula for calculating scores was as follows:

$$\mathrm{score }= \left(\mathrm{coefficient \, of \,Gene}\,1 \times \mathrm{ expression\, of \,Gene}\,1\right)+ \left(\mathrm{coefficient \,of \,Gene}\,2 \times \mathrm{expression \,of \,Gene}\,2\right)+\cdots + \left(\mathrm{coefficient\, of \,Gene \,n }\times \mathrm{ expression \,Gene\, n}\right)$$

Furthermore, to investigate the correlation between macrophage–naïve CD4 + T cell (MNT) score and overall survival, we performed survival analysis between the high and low-score groups using the “survival” package. We also performed gene set enrichment analysis (GSEA) to explore signaling pathways in the high-score group. Considering the IL16 related pathways activated between macrophages and naïve CD4 + T cells in cancer, we selected the potential signaling pathways in Molecular Signatures Database as input in GSEA. Regarding the immune cell infiltration in bulk transcriptomic data, we employed four algorithms (CIBERSORT, QUANTISEQ, MCP counter, and TIMER) to estimate and compare the difference between the high and low-score groups.

### Drug prediction

Drug sensitivity data of human cancer cell lines were obtained from the Cancer Therapeutics Response Portal (CTPR, https://portals.broadinstitude.org/ctrp) and PRISM Repurposing dataset (https://depmap.org/portal/prism/). The CTRP and PRISM contain the sensitivity data for 481 compounds over 835 CCLs and 1448 compounds over 482 CCLs, respectively. Both datasets employed the area under the dose–response curve (area under the curve, AUC) values to estimate drug sensitivity. Lower AUC values indicated higher sensitivity to treatment. For drug response prediction, we employed the ridge regression model located in the “pRRophetic” package to measure drug response and compared the difference in drug sensitivity between high and low-score groups. The algorithm of drug prediction was comprehensively described in previous study [23].

### Identification of Macrophage subtypes

Considering the various functions of macrophage subtypes, we investigated the correlation between identified macrophages (single-cell transcriptomic data) and macrophage infiltration (bulk transcriptomic data). We employed the differential expression genes of each state of macrophage to define the macrophage by ssGSEA. Considering the different states of macrophage, three datasets involved health (GSE33814), hepatitis (GSE33814), cirrhosis (GSE25097), and cancer (TCGA-LIHC) were using in this research. In terms of the subtypes of macrophage, we calculated the M1 polarization and M2 polarization score using ssGSEA. The defined genes were presented in Additional file 1: Table S2. Spearman method was used to calculate the correlation between different states of macrophage and polarization score.

### Spatial transcriptomic analyses

To further investigate the spatial location of single cell clusters in HCC TME, we employed “CARD” package [24] to deconvolute the spatial transcriptomic data based on our single cell data of cancer state. The spatial transcriptomic data were retrieved from previous study [25]. We selected four HCC tissue section for deconvolution. Using “CARD” package, we created “CARD” object through “CreateCARDObject” function. And we employed the “CARD_deconvolution” function with default parameters to calculate the results.

### Scissor analyses

To explore the underlying relationship between single cell clusters and clinical characteristics, our study used “Scissor” package [26] to analyze. Based on bulk transcriptomic data and phenotype information, “Scissor” can automatically identify cell subpopulations from single-cell data that are most responsible for the differences of phenotypes [26]. We employed TCGA-LIHC cohort and select “tumor size” and “stage” to analyze the single cell data of cancer. The “Scissor” function with “alpha = 0.01, family = binomial” parameters was executed.

## Results

### A multi-sector single-cell atlas of liver

**Fig. 1:**
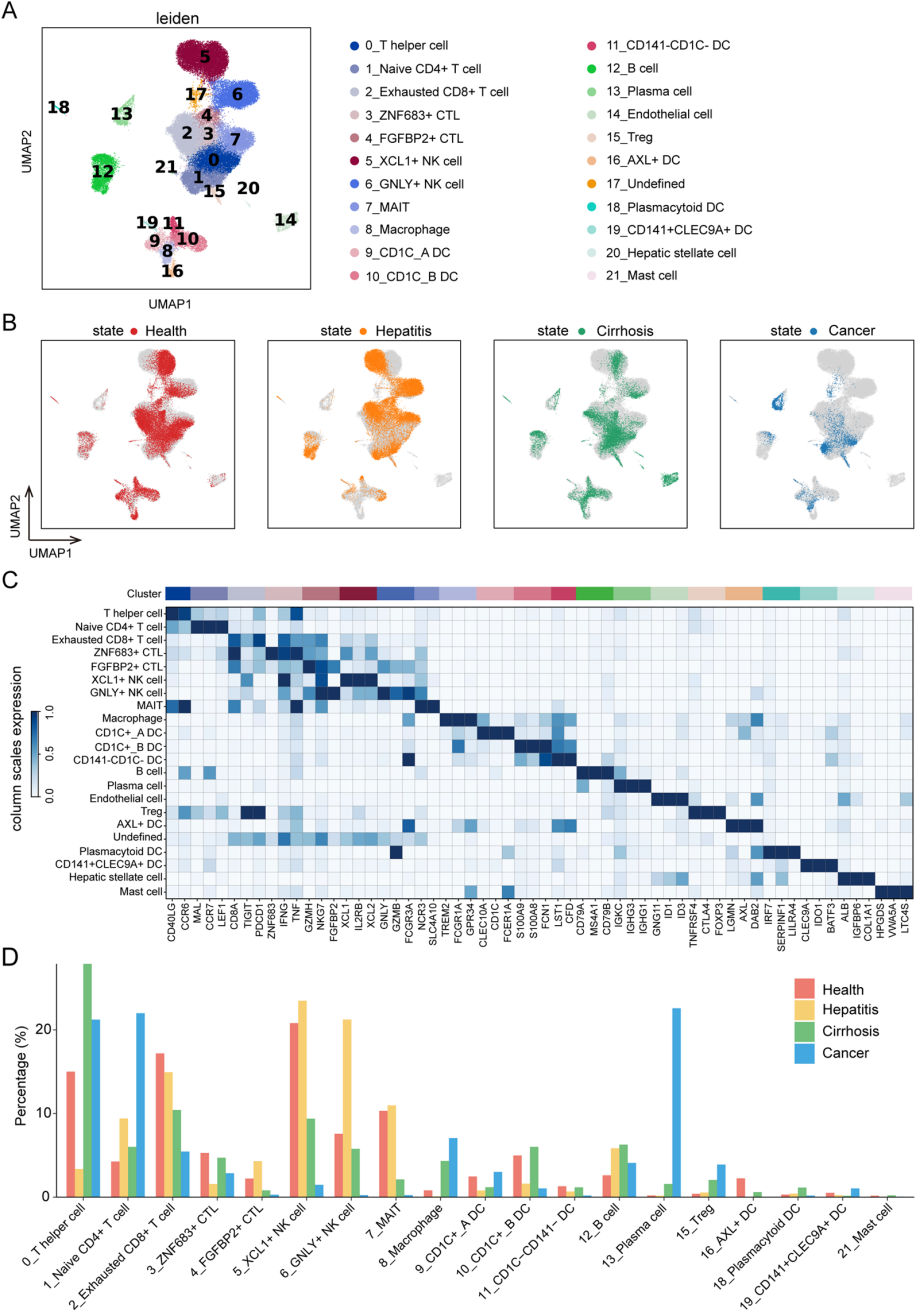
Identification of cell clusters and annotation. **A** Leiden clustering of 53,184 cells identifies 22 clusters. **B** UMAP plot colored by different states. **C** Dot plot depicting the cell-type-specific markers. **D** Proportion bar graph representing cluster frequency in four different states. In each state, the sum of each cluster proportion is 100%

In total, 53,184 cells from 31 cases were analyzed in our study. Leiden clustering of these cells identified 22 distinct major clusters representing epithelial, immune, endothelial, and fibroblast populations (Fig. 1A). Although we selected immune cells in data preprocessing, a fraction of stromal cells was found; this may be attributed to the different clustering methods and annotations. As illustrated in Additional file 1: Fig. S2G–J, the batch effects were removed. Figure 1B shows the distribution of different states in the UMAP. Next, we employed the specific markers to annotate leiden clusters into 21 cell types of the liver (cluster 17 was undefined), comprising T helper cells (CD40LG, CCR6), naïve CD4 + T cells (MAL, CCR7, LEF1), CD8 + exhausted T cells (CD8A, TIGIT, PDCD1), ZNF683 + cytotoxic T lymphocytes (ZNF683, IFNG, TNF), FGFBP2 + cytotoxic T lymphocytes (GZMK, NKG7, FGFBP2), XCL1 + natural killer cells (XCL1, IL2RB, XCL2), GNLY + natural killer cells (GNLY, GZMB, FCGR3A), mucosal-associated invariant T cells (NCR3, SLC4A10), macrophage (TREM2, FCGR1A,GPR34), CD1C + _A dendritic cells (CLEC10A, CD1C, FCER1A), CD1C + _B dendritic cells (S100A9, S100A8, FCN1), CD141-CD1C-dendritic cells (LST1, CFD), B cells (CD79A, MS4A1, CD79B), Plasma cells (IGKC, IGHG3, IGHG1), endothelial cells (GNG11, ID1, ID3), Treg cells (TNFRSF4, CTLA4, FOXP3), AXL + dendritic cells (LGMN, AXL, DAB2), plasmacytoid dendritic cells (IRF7, SERPINF1, LILRA4), CD141 + CLEC9A + dendritic cells (CLEC9A, IDO1, BATF3), Hepatic stellate cell (ALB, IGFBP6, COL1A1), and mast cells (HPGDS, VWA5A, LTC4S) (Fig. 1C and Additional file 1: Fig. S3). To characterize the change in each cell type among the four states, we calculated the proportion of cell types during each state. Interestingly, we observed that some immune cells have a relatively higher proportion in cancer, including T helper cells, naïve CD4 + T cells, exhausted CD8 + T cells, macrophages, B cells, plasma cells, and Treg cells (Fig. 1D). The statistical significance of each cell type in four states were presented in Additional file 1: Fig. S4.

**Fig. 2:**
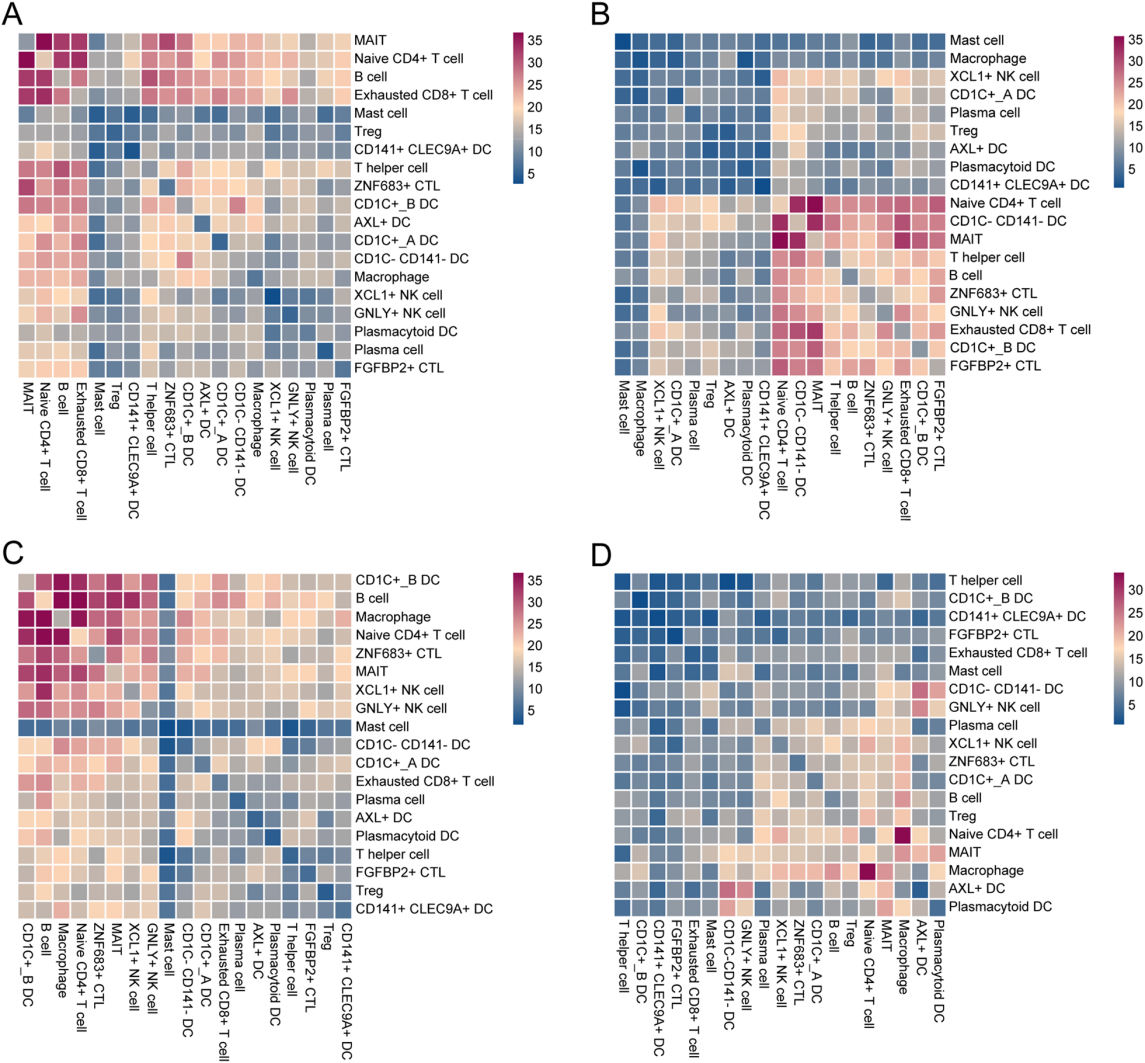
Cell interactions. The interactions among 19 cell types in **A** health, **B** hepatitis, **C** cirrhosis, and **D** cancer. Note: The scale number represents the interactions between cells. The higher number represents the stronger interactive strength

**Fig. 3:**
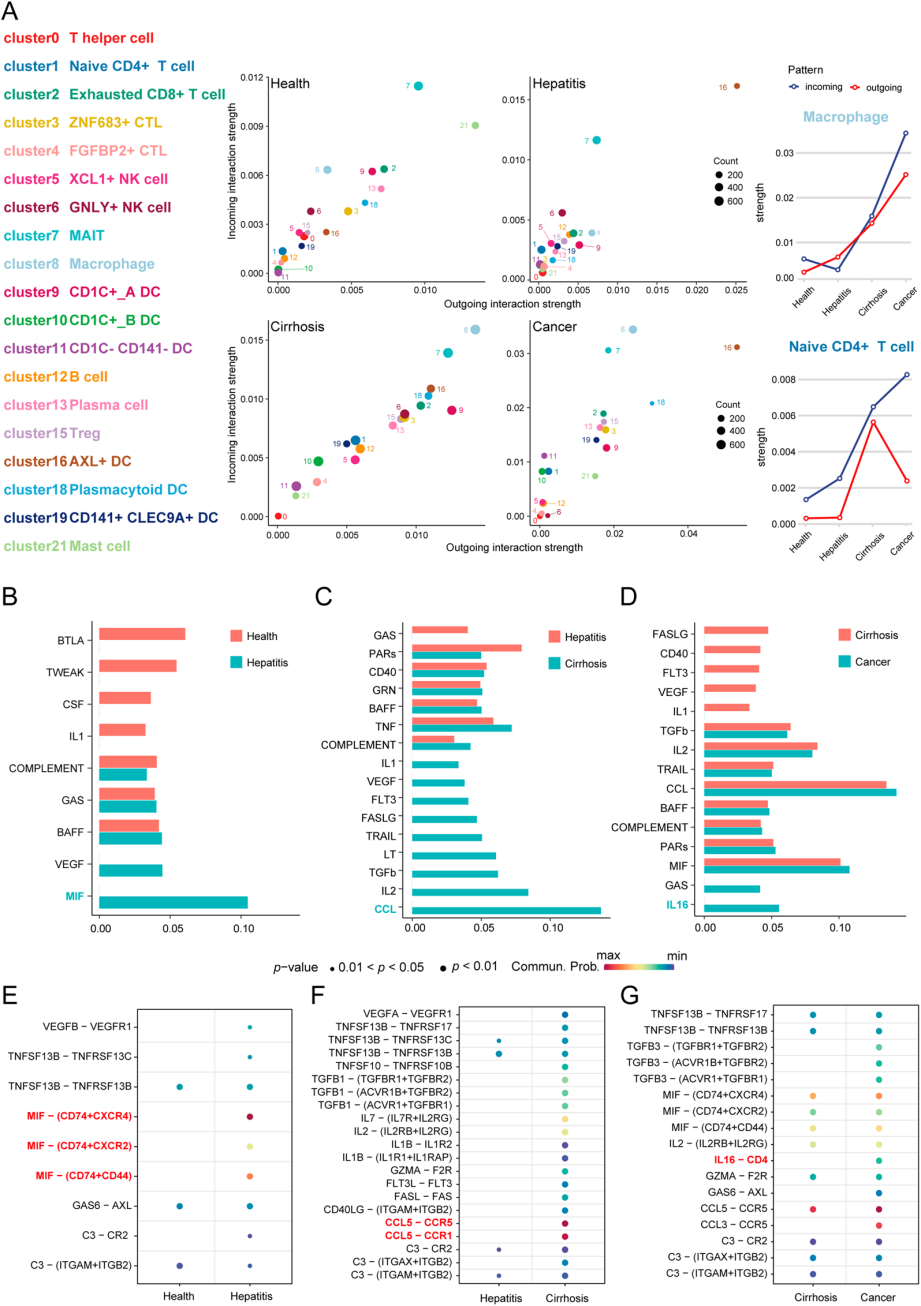
Strength and pathways involved in cell–cell interactions from four different states. **A** The incoming and outgoing strength of each immune cell under health, hepatitis, cirrhosis, and cancer, respectively. The communication probability of pathways from **B** health to hepatitis, **C** hepatitis to cirrhosis, and **D** cirrhosis to cancer. The difference in communication probability of ligand-receptor interactions from (**E** health to hepatitis, **F** hepatitis to cirrhosis, and **G** cirrhosis to cancer. Note: **B**–**G** is limited to the interaction of macrophages (sender) and naïve CD4 + T cells (receiver)

To decipher the ligand-receptor interactions in different states, we performed cell–cell interaction analyses. The results from CellPhoneDB indicated that different states exhibited various immune cell interactions (Fig. 2A–D). Furthermore, we observed that macrophage-naïve CD4 + T cell interaction had an important effect on the cirrhosis and cancer but not the hepatitis. Simultaneously, the results from CellChat revealed that macrophages exhibited higher outgoing interaction strength in cirrhosis and cancer, but lower strength in hepatitis (Fig. 3A). Meanwhile, naïve CD4 + T cells showed a lower outgoing interaction strength in health, hepatitis, and cancer states, but their outgoing strength increased in the cirrhosis state. We speculated that naïve CD4 + T cells may be regulated by macrophages through paracrine mechanisms, mainly in hepatitis, cirrhosis, and cancer. Consequently, we investigated the signaling pathways by which macrophages act as senders and naïve CD4 + T cells as receivers, in subsequent analyses. From the health to hepatitis, the MIF signaling network and related ligand-receptor interactions, including MIF-(CD74 + CXCR4), MIF-(CD74 + CXCR2), and MIF-(CD74 + CD44) showed a significant effect on macrophage–naïve CD4 + T cell interaction (Fig. 3B and E). From hepatitis to cirrhosis, the CCL signaling network and related ligand-receptor interactions, including CCL5-CCR5 and CCL5-CCR1, presented the highest communication probability in macrophage-naïve CD4 + T cell interaction (Fig. 3C and F). From the cirrhosis to cancer, IL16 and related ligand-receptor interaction (IL16-CD4) exhibited a significant difference in macrophage-naïve CD4 + T cell interaction (Fig. 3D and G).

**Fig. 4:**
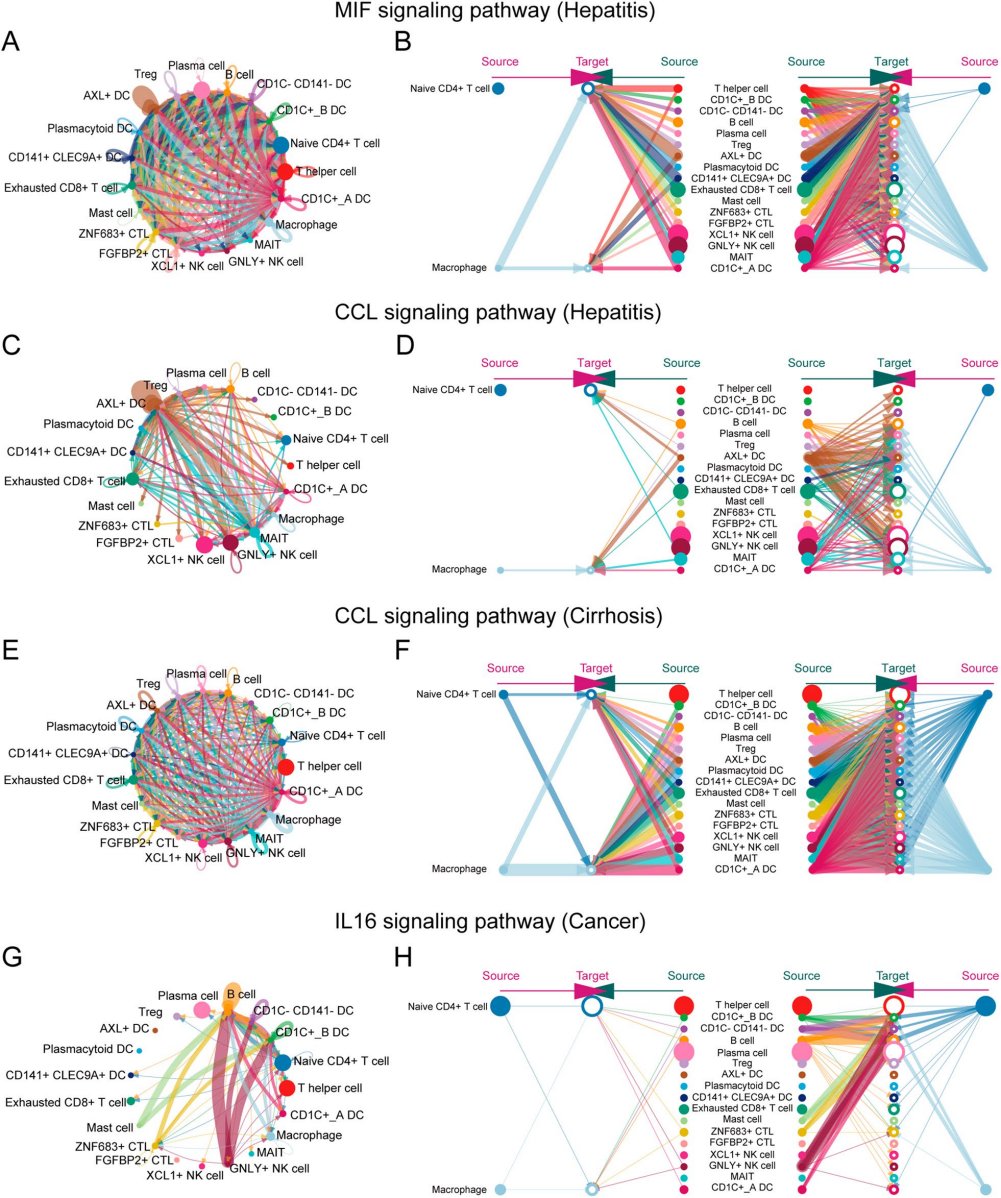
The cell–cell interactions in specific pathways under different states. **A** The MIF signaling pathway network in hepatitis. **B** The hierarchy plot of MIF signaling pathway in hepatitis. **C** The CCL signaling pathway network in hepatitis. **D** The hierarchy plot of CCL signaling pathway in hepatitis. **E** The CCL signaling pathway network in cirrhosis. **F** The hierarchy plot of CCL signaling pathway in cirrhosis. **G** The IL16 signaling pathway network in cancer. **H** The hierarchy plot of the IL16 signaling pathway in cancer

Furthermore, we visualized the network and interactions of the above-mentioned signaling pathways (MIF, CCL, and IL16). In the healthy state, the communication probability exhibited by the MIF signaling pathway was insignificant, similar to that of IL16 in the cirrhosis state. Therefore, the only difference observed was in the CCL signaling pathway, between hepatitis and cirrhosis in our study. As illustrated in Figs. 4A and B, the interactions based on the MIF signaling pathway between macrophages and other immune cells, were mediated mainly by paracrine signaling. From hepatitis to cirrhosis, the total interactions of the CCL signaling pathway increased significantly (Fig. 4C and E), and both the strength of autocrine and paracrine cells from macrophages and naïve CD4 + T cells, increased in the CCL signaling pathway (Fig. 4D and F). From cirrhosis to cancer, macrophages and naïve CD4 + T cells employ autocrine and paracrine mechanisms to communicate in the IL16 signaling pathway (Fig. 4G and H).

### Gene regulatory network in macrophages and naïve CD4 + T cells

**Fig. 5:**
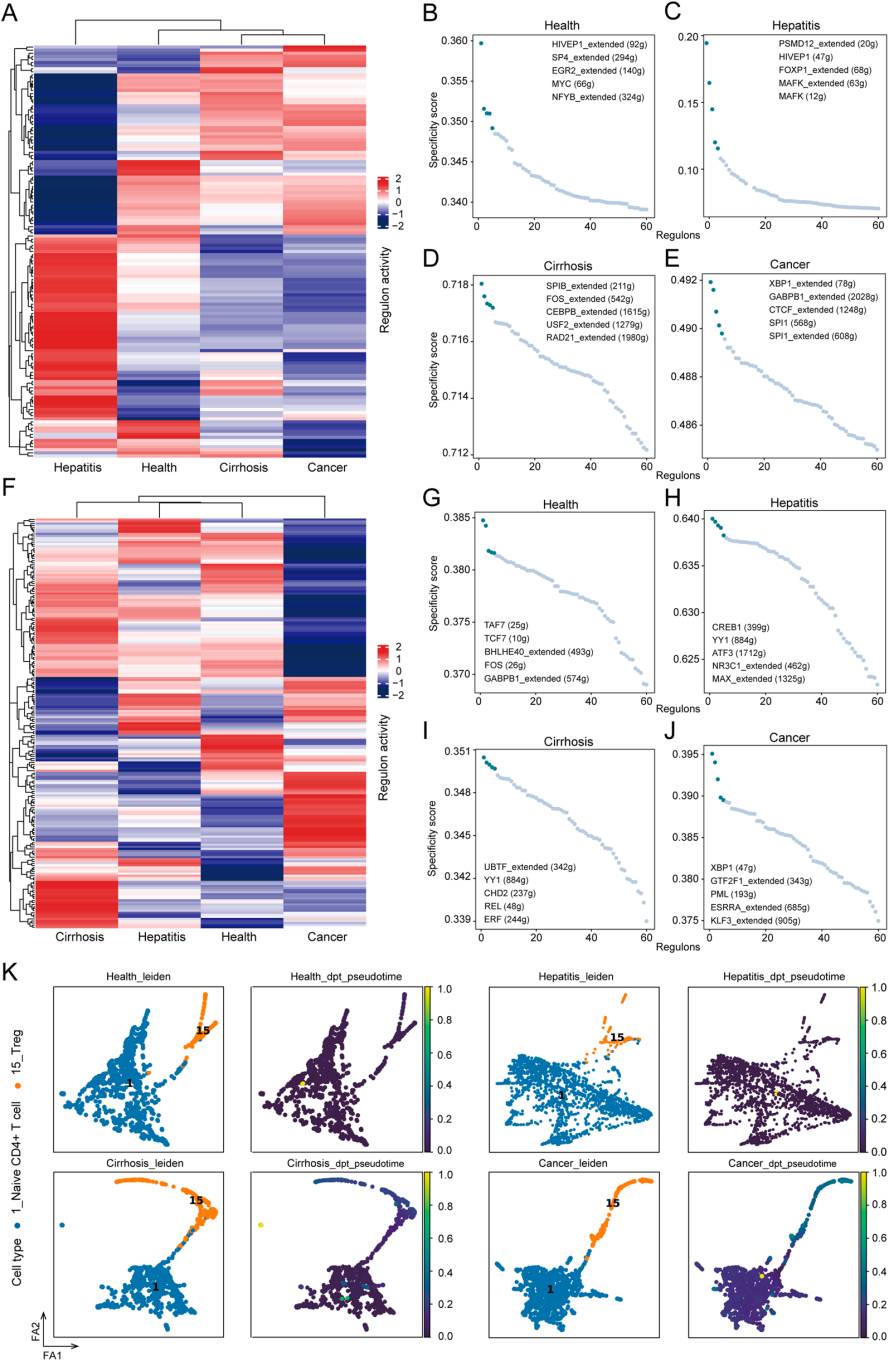
Gene regulatory network prediction and trajectory analysis. **A** Heatmap of macrophage-related regulons in different states. Top 5 regulons of macrophage in **B** health, **C** hepatitis, **D** cirrhosis, and **E** cancer. **F** Heatmap of naïve CD4 + T cell-related regulons in different states. Top 5 regulons of naïve CD4 + T cells in **G** health, **H** hepatitis, **I** cirrhosis, and **J** cancer. **K** Developmental trajectory of Treg cells and naïve CD4 + T cells in four different states inferred by PAGA, colored by cell types and pseudotime, respectively. Note: dpt means diffusion pseudo time. The scale represents the predictive differentiation trajectory. The higher value represents the degree of differentiation

Considering the importance of macrophage-naïve CD4 + T cell interaction in the TME, we employed SCENIC to decipher the gene regulatory network (GRN) of macrophages and naïve CD4 + T cells, followed by regulon-activity-based hierarchical clustering. The results indicated that cirrhosis and cancer presented similar GRN of macrophages, while the difference was observed between hepatitis and the other three states (Fig. 5A). Naïve CD4 + T cells exhibited distinct GRN in the cancer (Fig. 5F). Furthermore, we employed the RSS to evaluate regulons and ranked them in each state (Fig. 5B–E,G–J). We found that the transcriptomic factor (XBP1) was the top-ranked in both macrophages and naïve CD4 + T cells in cancer (Fig. 5E and J).

### Treg cells may derive from naïve CD4 + T cells in cancer

A previous study [27] revealed that Treg cells in human breast cancer are mainly derived from naïve CD4 + T cells recruited by tumor-associated macrophage-derived CCL18. Our results also demonstrated that Treg cells accounted for a relatively high proportion in the tumor microenvironment (Fig. 1D). We speculated that Treg cells may also be converted from naïve CD4 + T cells in cancer and thus, we performed a trajectory analysis. The results from PAGA validated our speculation (Fig. 5K). Furthermore, we found that naïve CD4 + T cells convert into Treg in specific states (cirrhosis and cancer), which may be related to macrophage.

### Validation from bulk transcriptomic data

#### Macrophage-naïve CD4 + T cell interaction score generation

**Fig. 6:**
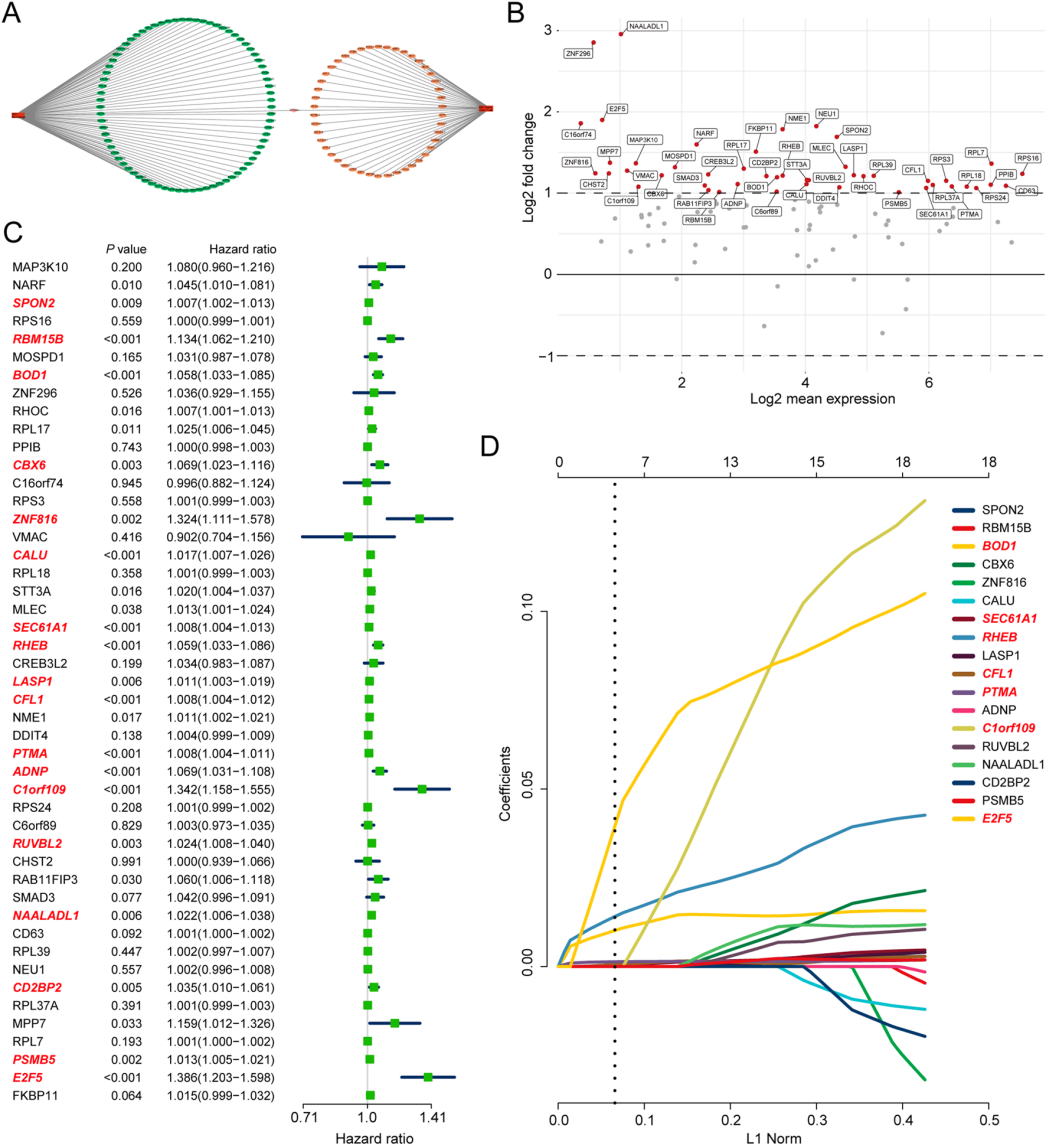
MNT score generation. **A** Top two regulon networks of macrophages and naïve CD4 + T cells in cancer. **B** Differentially expressed genes in TCGA cohort. **C** Forest plot of Cox regression analysis. **D** Results of lasso regression analysis

**Fig. 7:**
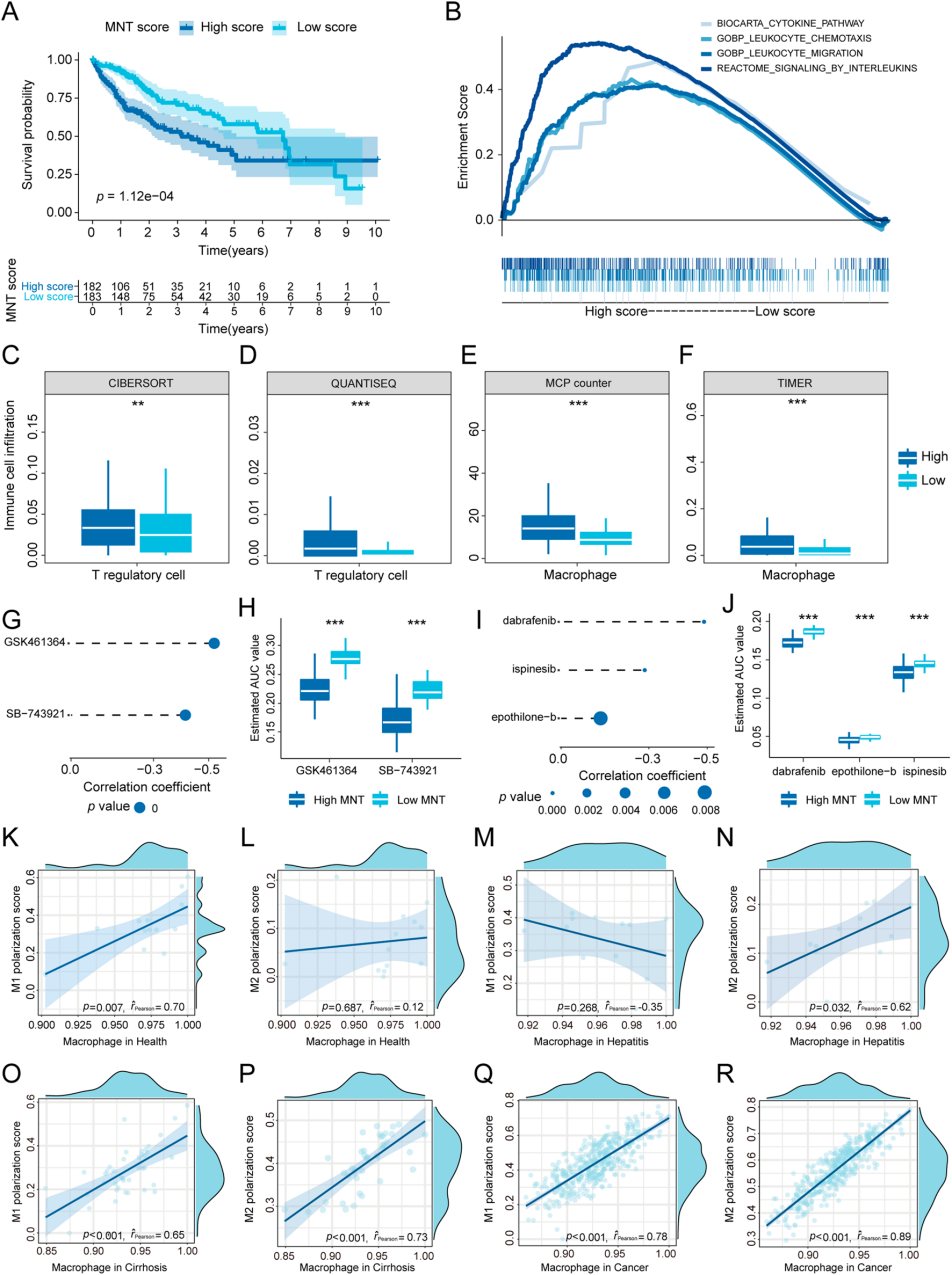
MNT score validation and drug prediction. **A** Survival analysis based on TCGA cohort. **B** Relevant pathways enriched in the high-score group. Treg cell infiltration differences between two groups based on **C** CIBERSORT, **D** QUANTISEQ, **E** MCP counter, and **F** TIMER. **G** Spearman’s correlation analysis of two CTPR-derived compounds. **H** Differential drug analysis of two CTPR-derived compounds. **I** Spearman’s correlation analysis of three PRISM-derived compounds. **J** Differential drug analysis of two PRISM-derived compounds. **K**, **L** Correlation analysis between macrophage (health) and **K** M1 polarization score, **L** M2 polarization score. **M**, **N** Correlation analysis between macrophage (hepatitis) and **M** M1 polarization score, **N** M2 polarization score. **O**, **P** Correlation analysis between macrophage (cirrhosis) and **O** M1 polarization score, **P** M2 polarization score. **Q**, **R** Correlation analysis between macrophage (cancer) and **Q** M1 polarization score, **R** M2 polarization score

To further elucidate the relationship between macrophage-naïve CD4 + T cell interaction and the clinic, we established a macrophage-naïve CD4 + T cell (MNT) score. First, we selected the target genes of the top regulon from macrophages (cancer) and naïve CD4 + T cells (cancer), respectively, and visualized them in network (Fig. 6A). A total of 125 target genes were utilized in our study, comprising 78 in macrophages (cancer) and 47 in naïve CD4 + T cells (cancer) (Additional file 1: Table S3). We then performed differential expression analysis of selected genes between the normal and tumor tissue based on TCGA-LIHC dataset. Forty-seven genes were eligible for Cox regression analysis (Fig. 6B). The results from the Cox regression analysis showed that 18 genes were significantly correlated with prognosis (*p* < 0.01) (Fig. 6C). Subsequently, we employed a LASSO regression model to establish the MNT score. The results from Fig. 6D revealed that the MNT score was generated by seven genes (BOD1, SEC61A1, RHEB, CFL1, PTMA, C1orf109, and E2F5). We divided the cases into high-score and low-score groups based on the median. A significant survival difference (*p* < 0.01) was found between the high and low-score groups (Fig. 7A). Another two HCC datasets (ICGC-LIRI and GSE54236) also demonstrated that the patients with higher MNT score presented the worst prognosis (Additional file 1: Fig. S5). The cases in the high-score group were significantly enriched in leukocyte-and cytokine-related pathways (Fig. 7B). We also found that the cases in the high-score groups presented higher infiltration of Treg cells and macrophages (Fig. 7C–F).

### Drug prediction based on CTPR and PRISM

We predicted the potential drugs based on the CTPR and PRISM databases (Fig. 7G–J) and found that the cases in the high-score group may be more sensitive to GSK461364 (inhibitor of polo-like kinase 1), SB-743921 (inhibitor of kinesin family member 11), dabrafenib, ispinesib, and epothilone-b.

### Macrophages in TME tend to exhibit the M2 phenotype

To further explore the function of macrophages in this study, we investigated the correlation between differential expression genes of macrophage and polarization score (M1, and M2). The results demonstrated that macrophage tended to present the M1 phenotype in health while M2 in hepatitis, cirrhosis, and cancer (Fig. 7K–R).

### Translational level validation from HPA database

To further validate the association between macrophage, naïve CD4 + T cell and Treg cell in liver carcinoma, we estimated the translational level of their marker genes using immunohistochemistry. Three maker genes were found in liver carcinoma of HPA database (TREM2 and GPR34 from macrophage, PDCD1 from Treg cell), which indicated that macrophages and Treg cells existed in liver carcinoma (Additional file 1: Fig. S6).

### Spatial co-location was observed between Macrophage and Treg

The deconvolution results of HCC spatial transcriptomic data indicated that macrophage and Treg existed in HCC tissues (Additional file 1: Fig. S7). Furthermore, we observed that both macrophage and Treg located in the similar position (Additional file 1: Fig.S7), which indicated that there was higher communication probability between macrophage and Treg in cancer.

### Macrophage, naïve CD4 + T cell and Treg associated with HCC progression

To further explore the association between cell types and clinical characteristics, we combined the bulk transcriptomic data (TCGA-LIHC) and single-cell transcriptomic data (cancer) to performed scissor analyses. The results showed that 80.4% cells in cancer associated with T3/T4, in which naïve CD4 + T cell accounts for 21.5%, macrophage accounts for 11.5% and Treg accounts for 5% (Additional file 1: Fig. S8). Regarding the stage, 77% cells in cancer associated with stage III/stage IV, including 22.8% of naïve CD4 + T cell, 13.9% of macrophage and 4% of Treg (Additional file 1: Fig. S8).

## Discussion

Primary liver carcinoma commonly arises in damaged liver and is characterized by extensive inflammation and fibrosis. The TME, including immune cells, reacts to liver injury by producing cytokines and components of the extracellular matrix, which promotes angiogenesis and survival of cancer stem cells [28]. Generally, primary liver carcinoma presents an immunosuppressive microenvironment. Nevertheless, the correlation between the immunosuppressive microenvironment and liver injury (hepatitis and cirrhosis) remains unclarified. Here, we generated a comprehensive single-cell atlas of the liver to understand the immune cell interactions among health, hepatitis, cirrhosis, and cancer. We first focused on the proportion of each immune cell type in the four states and found that macrophages account for a relatively high proportion of tumor microenvironments. Simultaneously, macrophages and naïve CD4 + T cells exhibit stronger interactions in cancer, which indicated that macrophage-naïve CD4 + T cell interaction essentially affects the cancerous state. These results led us to investigate the differences of macrophage-naïve CD4 + T cell interaction in the four states. Notably, macrophages present higher outgoing and incoming strength in cirrhosis and cancer but not in hepatitis, possibly due to the decreased number of macrophages in hepatitis. Interestingly, we found that MIF-related ligand-receptor interactions were highly activated in hepatitis. MIF exhibits a protective effect against steatosis, resulting in a decreased quantity of macrophages [29]. The interaction between CCL5 and CCR5 significantly influences macrophage numbers from the state of hepatitis to cirrhosis. The receptor CCR5 induces the recruitment of macrophages [30]; thus, more macrophages can be observed in cirrhosis. A previous study found that the ligand CCL5 promotes steatosis, inflammation [31], and early cirrhosis [32]. The ligand-receptor pair of IL16 and CD4 is activated in the interaction of macrophages and naïve CD4 + T cells, and in conversion from cirrhosis to the cancer. IL16 induces the recruitment of macrophages in breast cancer [33]. The above-mentioned results elucidated the differences of macrophage-naïve CD4 + T cell interaction under the four states, which may be helpful in exploring the source of immunosuppressive microenvironment of liver carcinoma.

Next, we predicted the GRNs of macrophages in the four states. Importantly, the GRNs of macrophages between cirrhosis and cancer were similar, indicating that tumor-associated macrophages may stem from a cirrhotic state. Macrophages in hepatitis presented a distinct GRN compared to the other three states, revealing that macrophages may perform different functions in hepatitis. Besides, the identified macrophages tended to exhibit the M2 phenotype, which demonstrates that macrophages have an immunosuppressive effect in cancer. Furthermore, a previous study reported that naïve CD4 + T cells are recruited and converted to Treg cells by macrophages-derived CCL18 in breast cancer [27]. In our trajectory analyses of four states, the phenomenon that naïve CD4 + T cell differentiated into Treg occurred first in cirrhosis and then became more obvious in cancer, which was unobserved in health and hepatitis. Interestingly, the interaction between macrophage and naïve CD4 + T cell exhibited in CellphoneDB is consistent with the findings of trajectory analyses. The consistency further confirms the connection between the macrophage-naive CD4 + T cell interaction and Treg population. In summary, macrophages produced in response to cirrhosis play a crucial role in constructing and enhancing the immunosuppressive microenvironment of primary liver carcinoma.

Considering the limited therapy available for primary liver carcinoma, we predicted potential sensitive drugs based on macrophage-naïve CD4 + T cell interaction. First, we generated the MNT score to quantify macrophage-naïve CD4 + T cell interaction. For all cases, high MNT scores led to a worse prognosis, validating the importance of macrophage-naïve CD4 + T cell interaction in bulk transcriptomic data. Meanwhile, the cases with high MNT scores presented higher infiltration of Treg cells and macrophages, which corresponds with the results of single-cell analyses. Regarding drug prediction, inhibitors of polo-like kinase 1 and kinesin 11 based on CTPR, were significantly correlated with high MNT scores. Polo-like kinase 1 has been shown to regulate liver tumor cell death by phosphorylation of Tap63 [34]. Meanwhile, kinesin family member 11 has been shown to correlate with tumor size and prognosis in liver cancer [35]. Three other drugs from PRISM also cause concern. Dabrafenib is a single-agent treatment for patients with BRAF V600E mutation-positive advanced melanoma [36]. Epothilone-b is an anti-cancer drug for treating aggressive metastatic or locally advanced breast cancer, and prevents cancer cells from dividing by interfering with tubulin [37]. Interestingly, ispinesib is also an inhibitor of KIF11, which has been used to treat patients with locally advanced, recurrent, or metastatic liver cancer (NCT00095992). We reasoned that employing the predictive drugs may target the interaction of macrophages and CD4 naïve T cells, which results in reprogramming of the TME and strengthening of immunosurveillance in primary liver carcinoma.

Compared with the previous studies [6, 10, 11], our research integrated the single cell transcriptomic data of four states from healthy liver to primary liver carcinoma, which was comprehensive and creative. By comparing the TME of four states, we found that different states presented the vary proportion and communication among immune cells. The key point we found in this research was the interaction of macrophage and naïve CD4 + T cell. Macrophage induced naïve CD4 + T cell to differentiate into Treg in cirrhosis and cancer. Simultaneously, we observed that Macrophage and Treg co-located in HCC tissue. These findings may be helpful in decoding the immunosuppressive TME of HCC under single cell resolution. Furthermore, we linked the single cell transcriptomic and clinical phenotypes and verified the clinical importance of Macrophage. At present, macrophage-related therapy in HCC mainly divided into three approaches, including cutting off the source and eliminating the production of M2 macrophages, remodeling M2 macrophages to M1 macrophages, and blocking communication between M2 macrophages and liver cancer cells [38]. Blocking the interaction of macrophage and naïve CD4 + T cell may be the novel and interesting therapeutic strategy in HCC immunotherapy.

## Conclusion

In conclusion, this study reveals the crucial role of macrophage-naïve CD4 + T cell interaction in the immunosuppressive microenvironment of primary liver carcinoma. Tumor-associated macrophages may derive from cirrhosis. Naïve CD4 + T cell induced by macrophage may differentiate into Treg in cirrhosis and they finally contributed to immunosuppressive TME. Predictive drugs that target the macrophage-naïve CD4 + T cell interaction may help to improve the immunosuppressive microenvironment and prevent immune evasion. However, due to the bioinformatics used in our research, the relevant mechanisms need to further validations by experiments and cohort studies.

## Supplementary Information

**Additional file 1: Figure S1.** The workflow of study. **Figure S2.** Three violin plots of the computed quality measures: (A) the number of genes expressed in the count matrix, (B) the total counts per cell, and (C) the percentage of counts in mitochondrial genes. Two scatter plots: (D) the number of genes expressed in the count matrix and the total counts per cell, (E) the percentage of counts in mitochondrial genes and the total counts per cell. (F) The result of principal component analysis. This gives us information about how many PCs we should consider in order to compute the neighborhood relations of cells. The umap of four states before (G) and after (H) batch effect removing. The umap of three datasets before (I) and after (J) batch effect removing. **Figure S3.** The marker genes presented in umap. **Figure S4.** Statistical significance between four states in each cell type. Note: each circle represents the sample. **Figure S5.** Validation of survival analyses based on MNT score from ICGC-LIRI and GSE54236 cohorts. **Figure S6.** The translational level of TREM2, GPR34, and PDCD1 in immunohistochemistry. **Figure S7.** Deconvolution of spatial transcriptomic data. Note: The higher value represents the higher probability of the target cell type location. **Figure S8.** The association between cells from cancer and clinical characteristics (tumor size and stage). The left umaps were the results of Scissor analyses. The right bar plots were the proportion of each cell type in Scissor+ cell group. **Table S1.** The details of three public datasets. **Table S2**. The representative genes of M1 and M2 polarization. **Table S3. **The transcription factors of top 1 regulons from macrophage and naïve CD4+ T cells in cancer.

## Data Availability

The datasets generated during the current study are available in the TCGA database (https://portal.gdc.cancer.gov/), GEO database (https://www.ncbi.nlm.nih.gov/geo/) and ICGC database (https://dcc.icgc.org/).
