# Supplementary material for: Single-cell transcriptomics reveals the role of Macrophage-Naïve CD4 + T cell interaction in the immunosuppressive microenvironment of primary liver carcinoma
Source: J Transl Med. 2022 Oct 11;20:466. doi: 10.1186/s12967-022-03675-2 (PMC9552358; doi:10.1186/s12967-022-03675-2)
Supplement: Supplementary file 1 — Additional file 1: Figure S1. The workflow of study. Figure S2. Three violin plots of the computed quality measures: (A) the number of genes expressed in the count matrix, (B) the total counts per cell, and (C) the percentage of counts in mitochondrial genes. Two scatter plots: (D) the number of genes expressed in the count matrix and the total counts per cell, (E) the percentage of counts in mitochondrial genes and the total counts per cell. (F) The result of principal component analysis. This gives us information about how many PCs we should consider in order to compute the neighborhood relations of cells. The umap of four states before (G) and after (H) batch effect removing. The umap of three datasets before (I) and after (J) batch effect removing. Figure S3. The marker genes presented in umap. Figure S4. Statistical significance between four states in each cell type. Note: each circle represents the sample. Figure S5. Validation of survival analyses based on MNT score from ICGC-LIRI and GSE54236 cohorts. Figure S6. The translational level of TREM2, GPR34, and PDCD1 in immunohistochemistry. Figure S7. Deconvolution of spatial transcriptomic data. Note: The higher value represents the higher probability of the target cell type location. Figure S8. The association between cells from cancer and clinical characteristics (tumor size and stage). The left umaps were the results of Scissor analyses. The right bar plots were the proportion of each cell type in Scissor+ cell group. Table S1. The details of three public datasets. Table S2. The representative genes of M1 and M2 polarization. Table S3. The transcription factors of top 1 regulons from macrophage and naïve CD4+ T cells in cancer. [file 12967_2022_3675_MOESM1_ESM.docx]

**Supplementary Material**


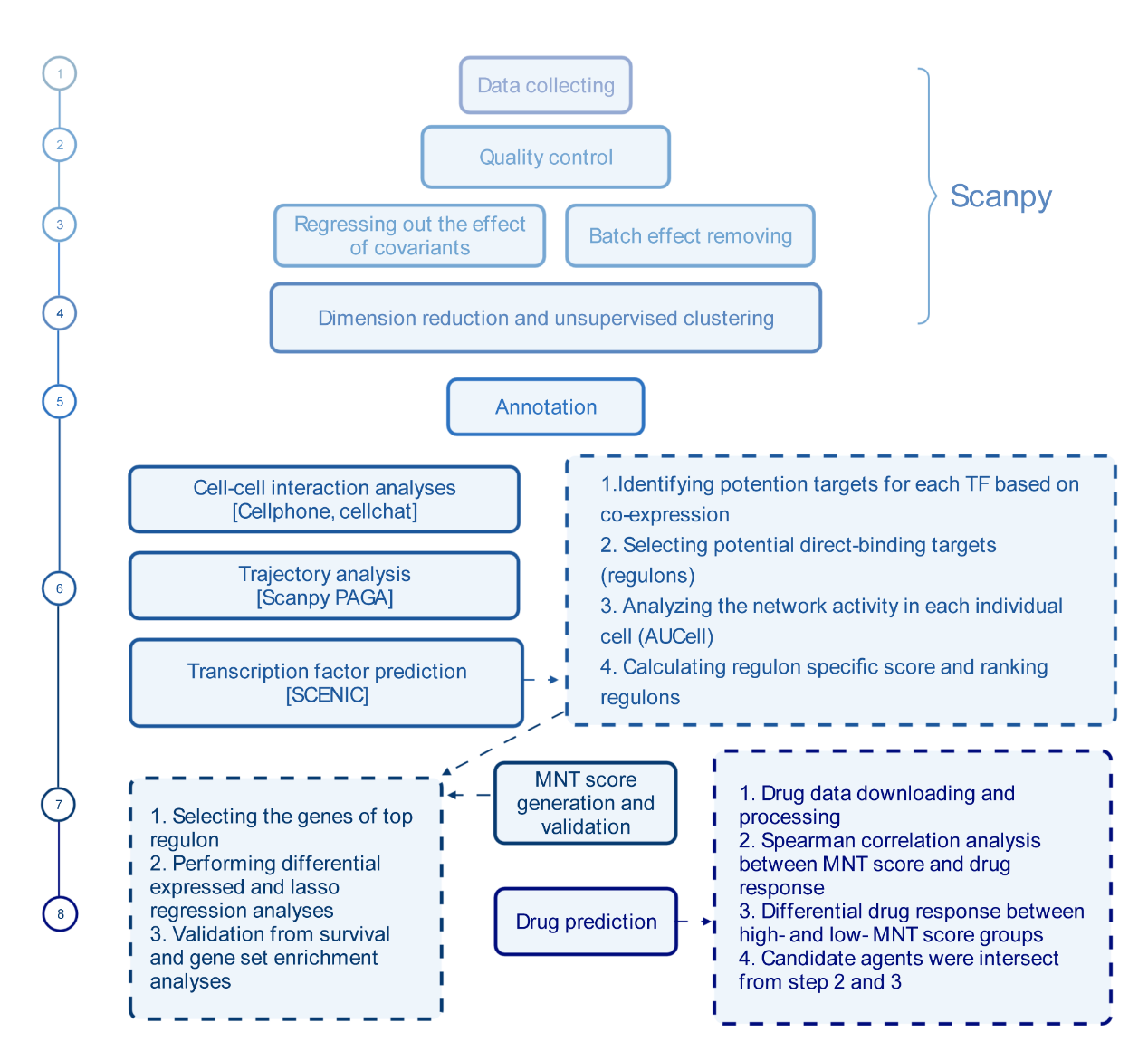


Figure S1. The workflow of study.


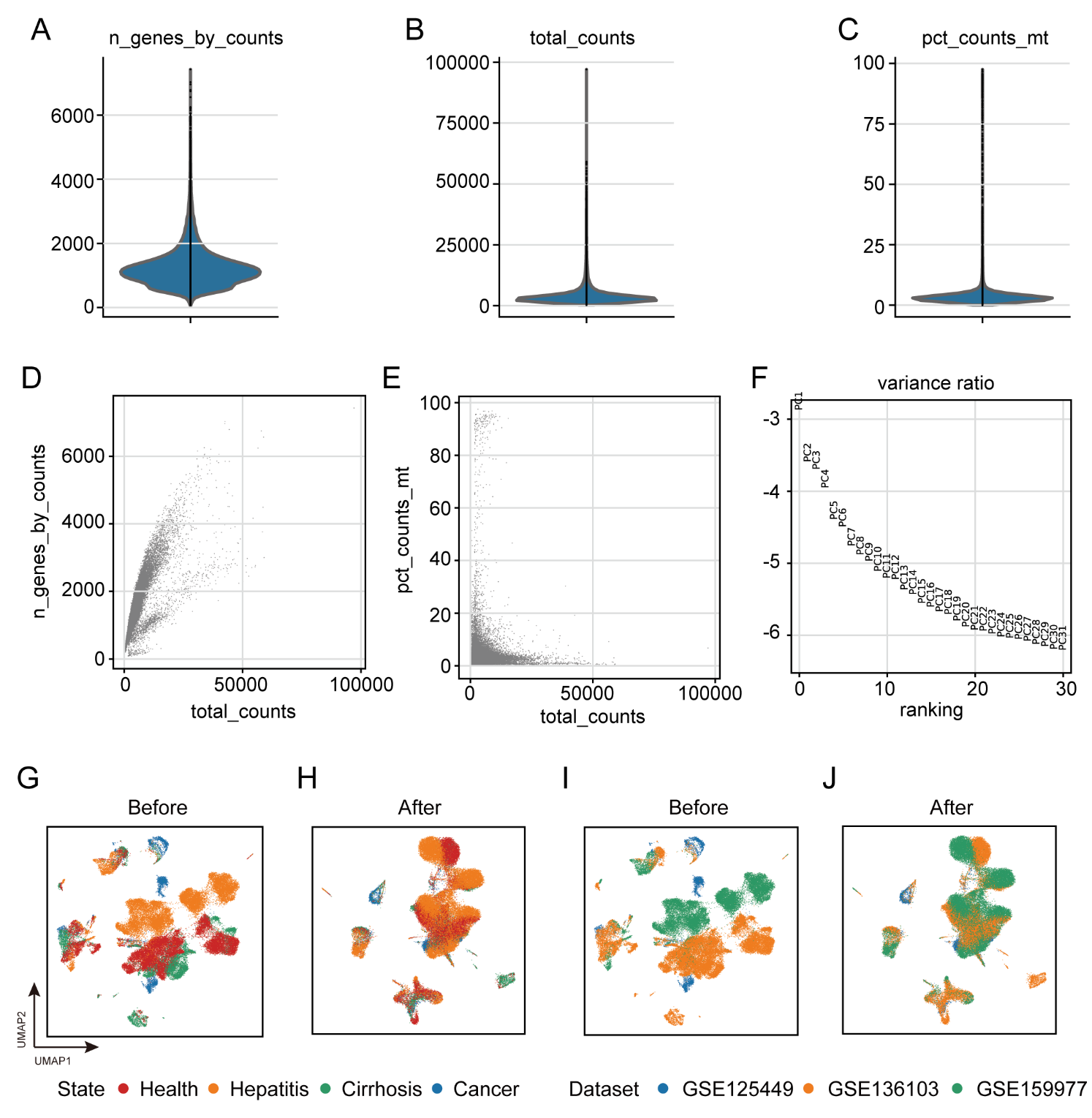


Figure S2. Three violin plots of the computed quality measures: (A) the number of genes expressed in the count matrix, (B) the total counts per cell, and (C) the percentage of counts in mitochondrial genes. Two scatter plots: (D) the number of genes expressed in the count matrix and the total counts per cell, (E) the percentage of counts in mitochondrial genes and the total counts per cell. (F) The result of principal component analysis. This gives us information about how many PCs we should consider in order to compute the neighborhood relations of cells. The umap of four states before (G) and after (H) batch effect removing. The umap of three datasets before (I) and after (J) batch effect removing.


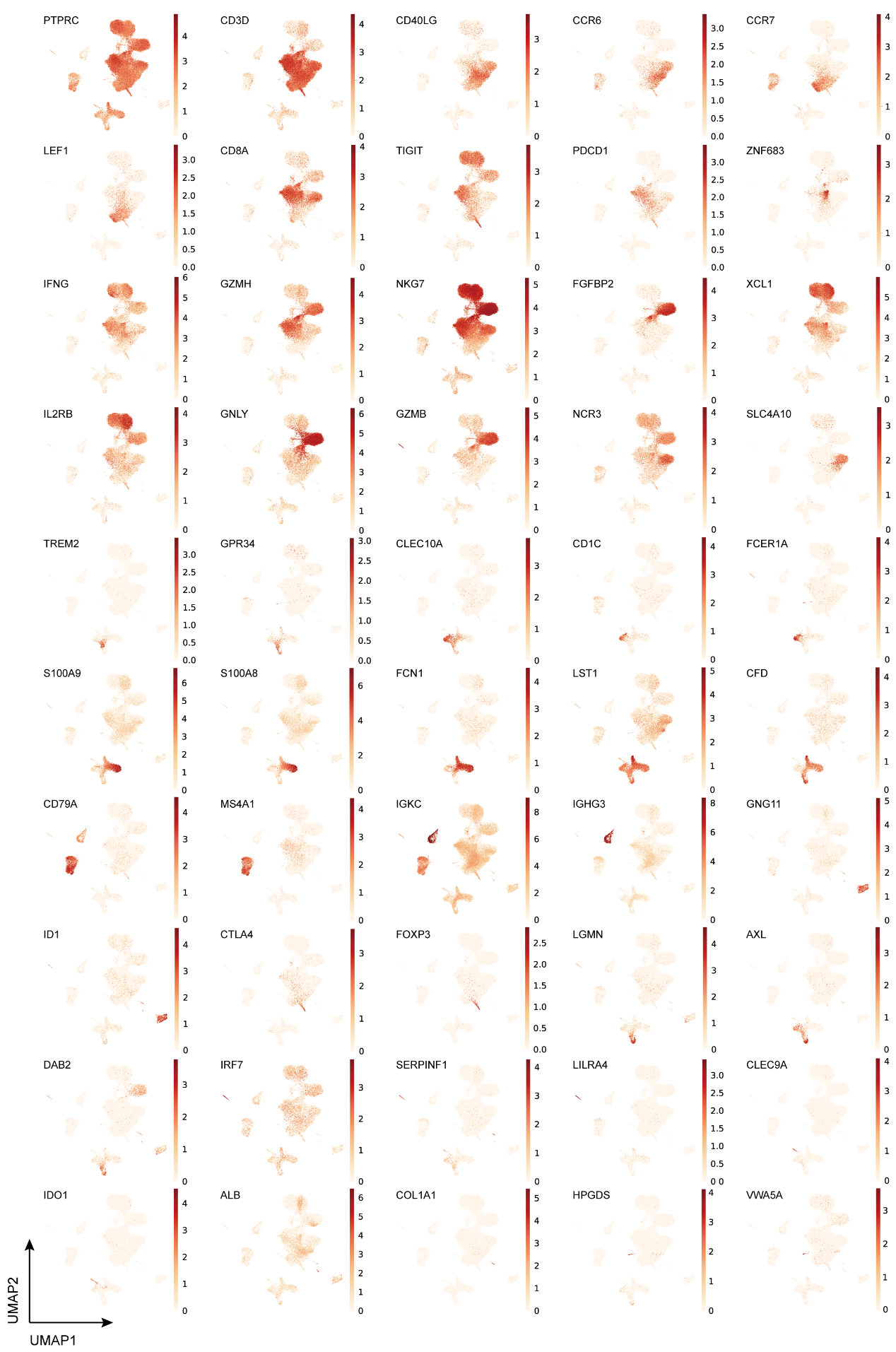


Figure S3. The marker genes presented in umap.


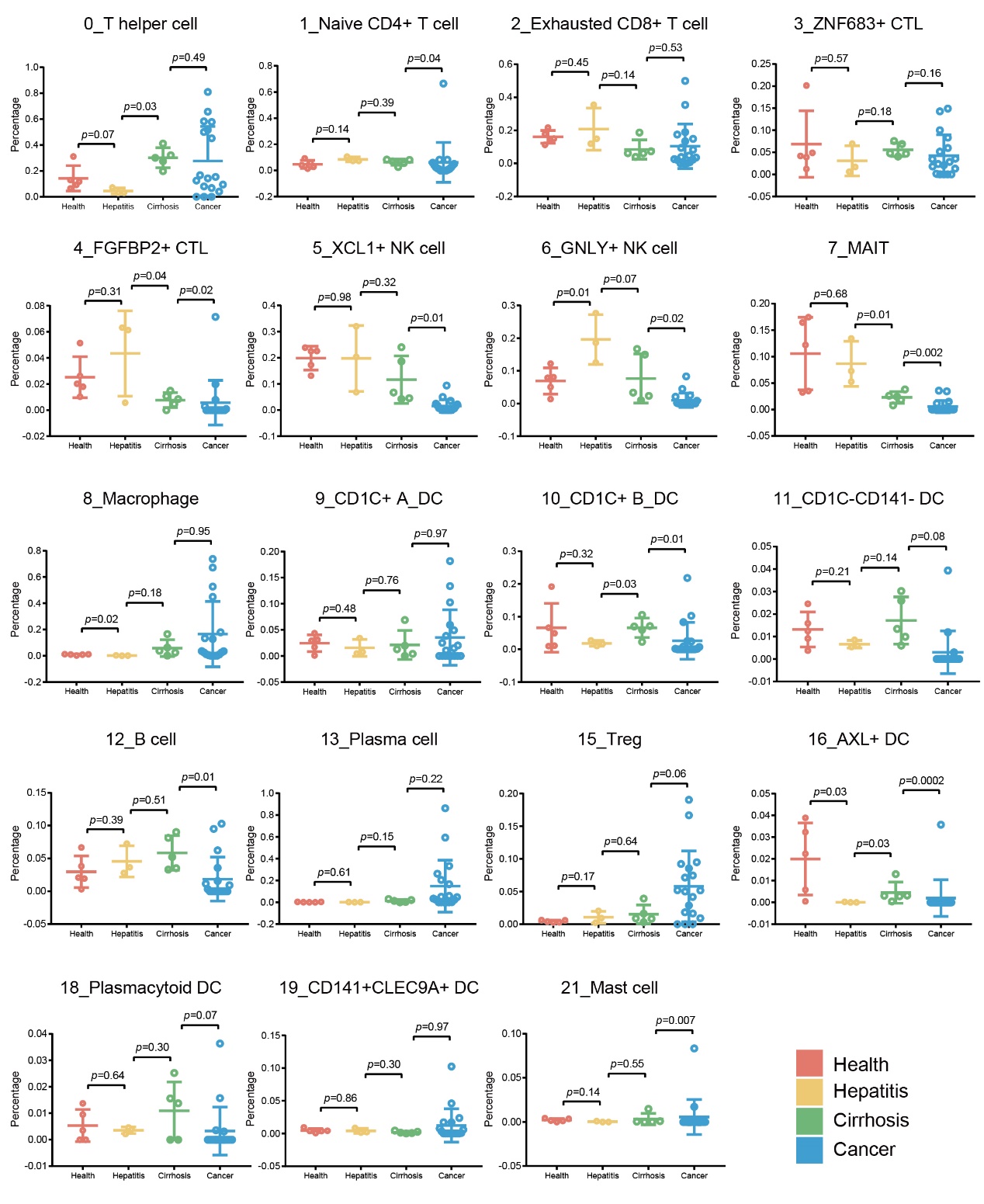


Figure S4. Statistical significance between four states in each cell type. Note: each circle represents the sample.


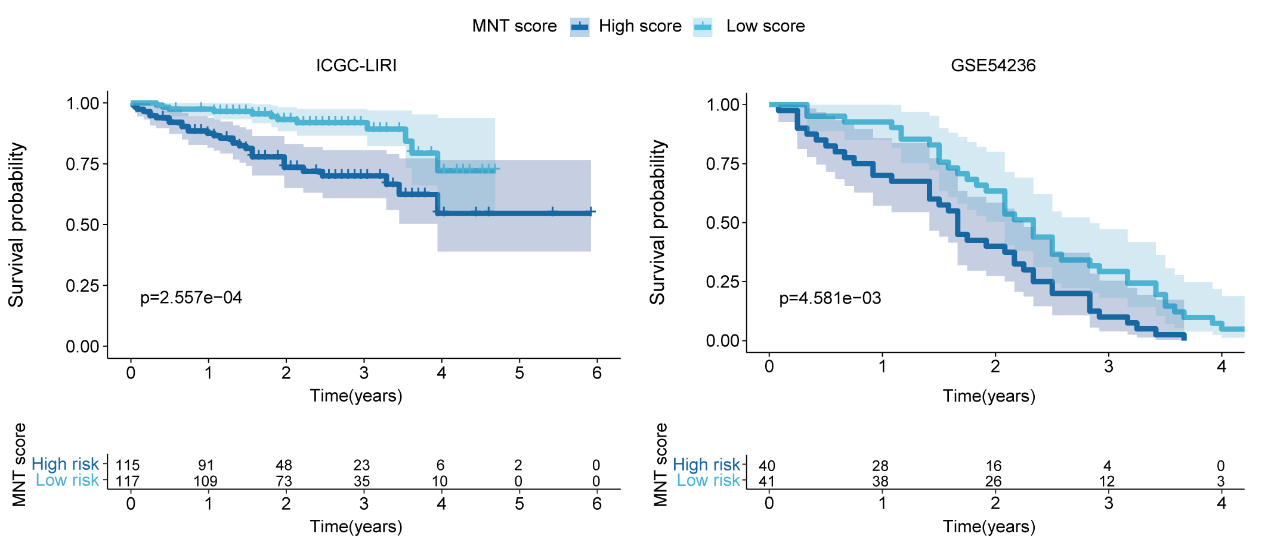


Figure S5. Validation of survival analyses based on MNT score from ICGC-LIRI and GSE54236 cohorts.


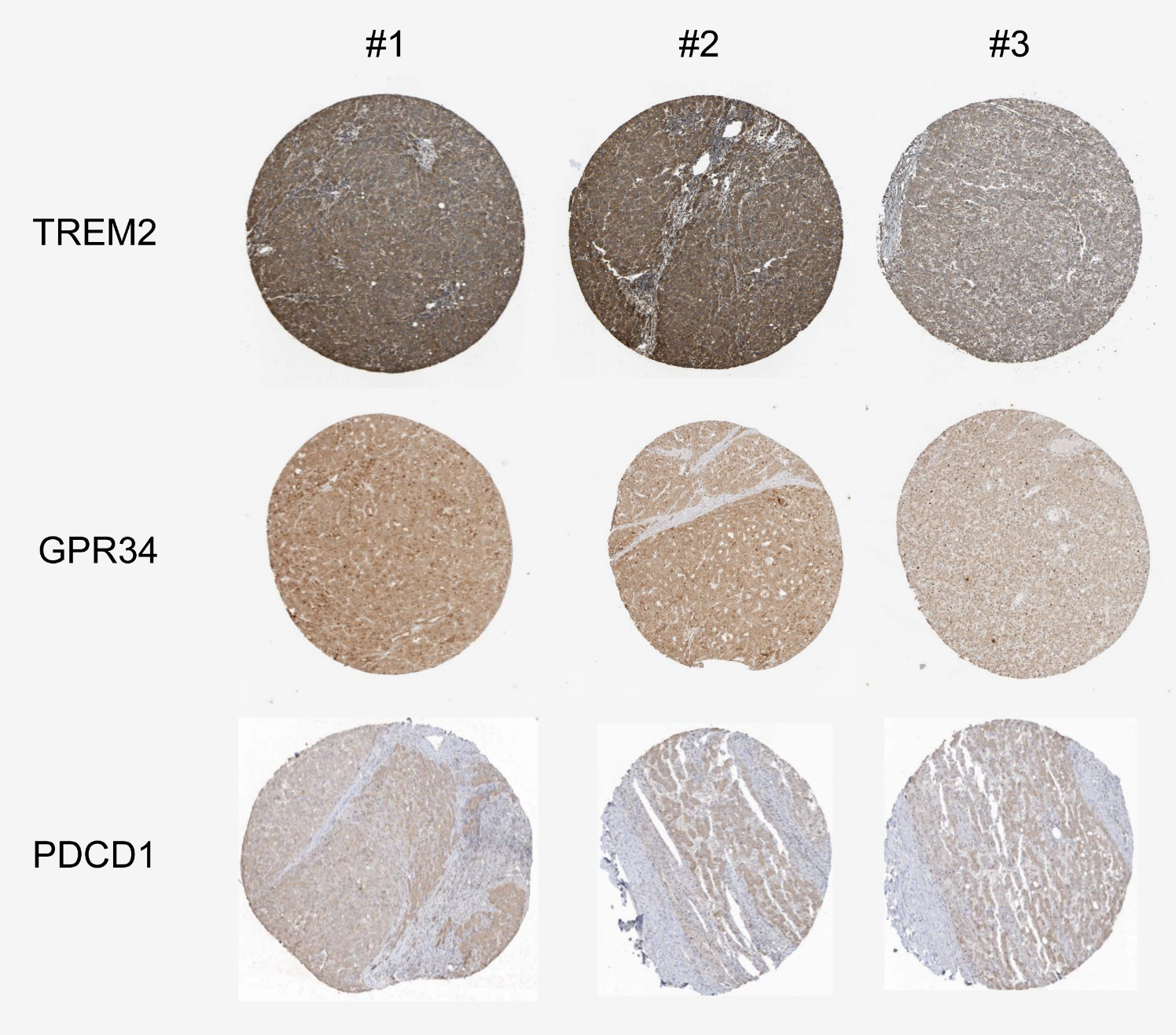


Figure S6. The protein level of TREM2, GPR34, and PDCD1 in immunohistochemistry.


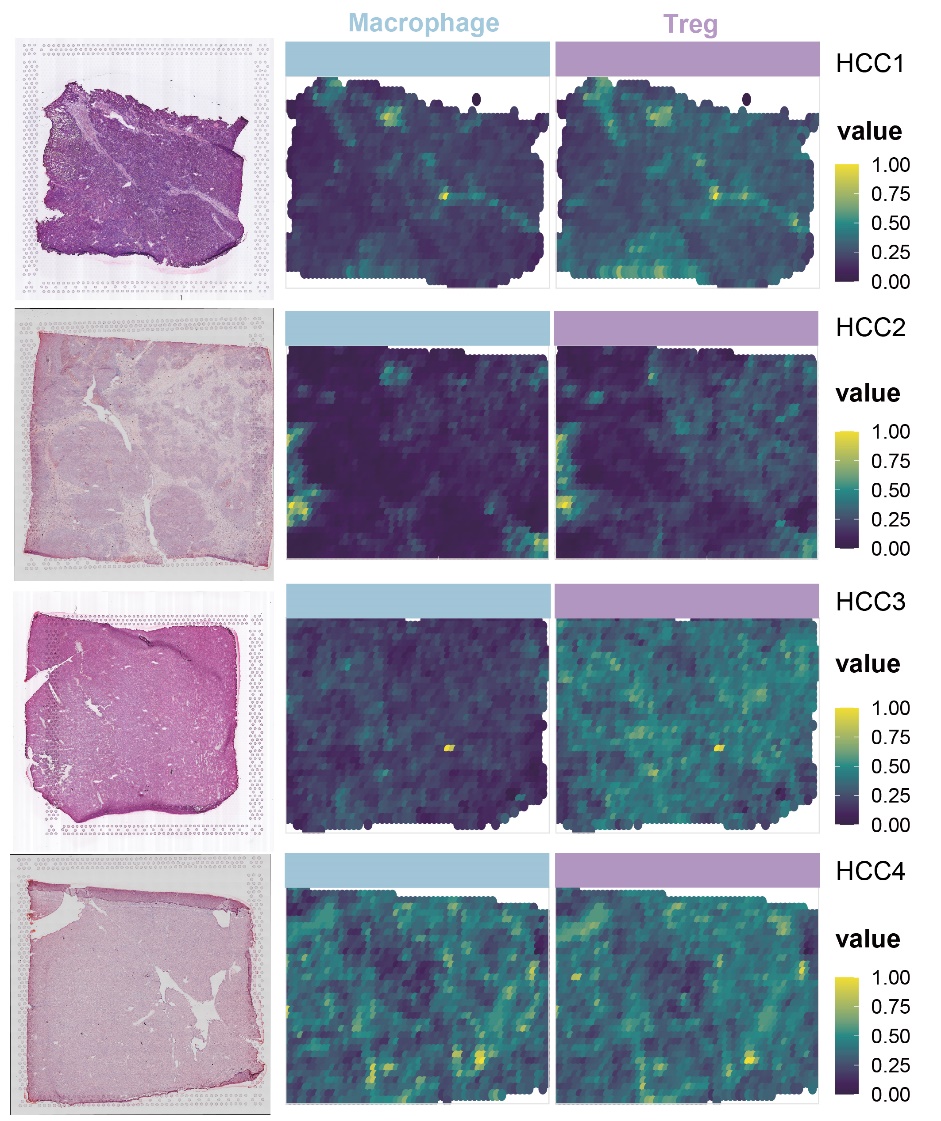


Figure S7. Deconvolution of spatial transcriptomic data. Note: The higher value represents the higher probability of the target cell type location.


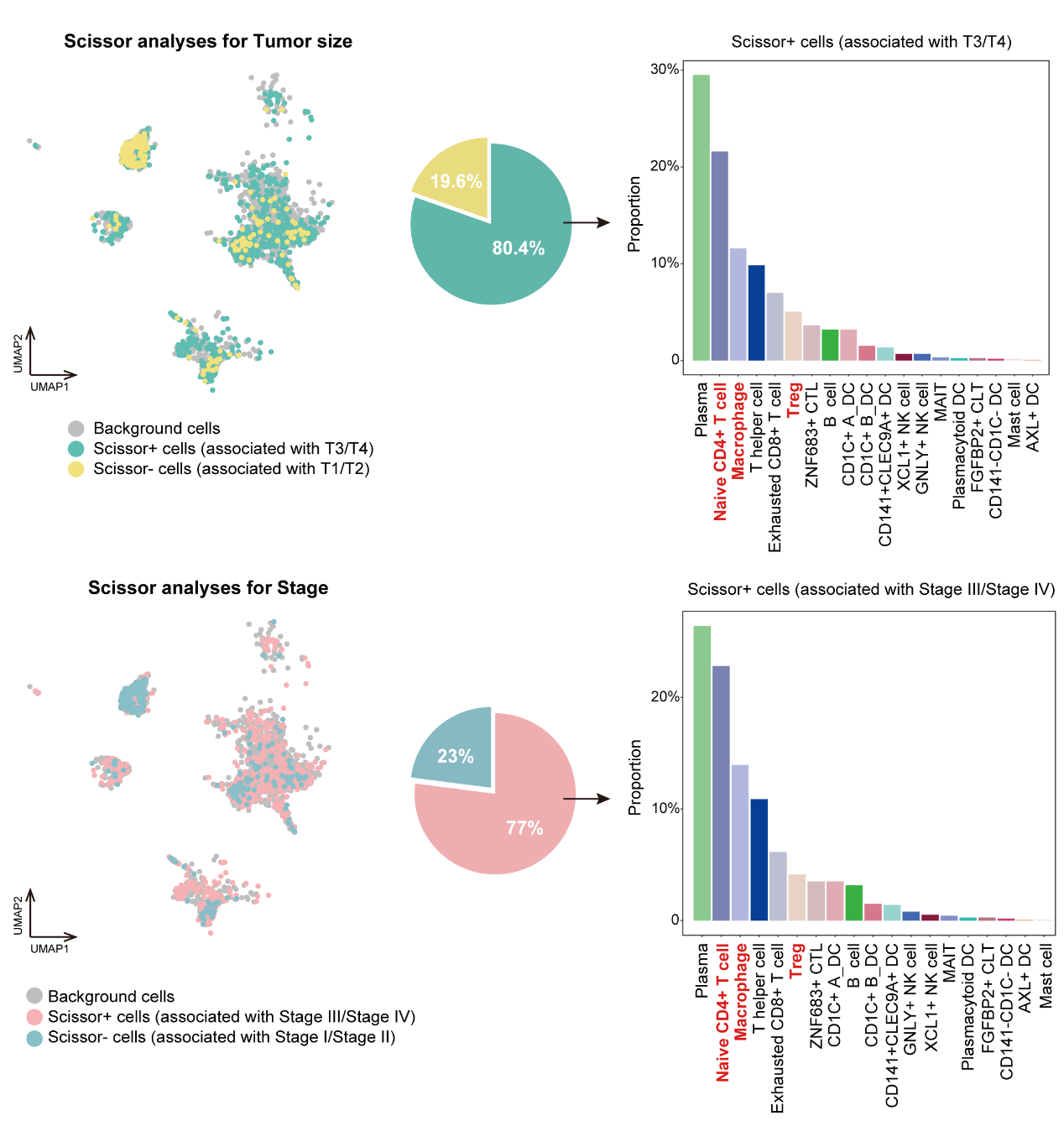


Figure S8. The association between cells from cancer state and clinical characteristics (tumor size and stage). The left umaps were the results of Scissor analyses. The right bar plots were the proportion of each cell type in Scissor+ cell group.

**Table S1. The details of three public datasets.**

| **Series** | **Platform** | **Samples** | **Status** | **Cell number** | **Sex** | **Age** | **Race** |
| --- | --- | --- | --- | --- | --- | --- | --- |
| GSE  159977 | 10X  Genomics | [GSM4851988](https://www.ncbi.nlm.nih.gov/geo/query/acc.cgi?acc=GSM4851988) | Hepatitis (NASH) | 12024 | NA | | |
|  |  | [GSM4851994](https://www.ncbi.nlm.nih.gov/geo/query/acc.cgi?acc=GSM4851994) | Hepatitis (NASH) | 6327 |  |  |  |
|  |  | [GSM4851996](https://www.ncbi.nlm.nih.gov/geo/query/acc.cgi?acc=GSM4851996) | Hepatitis (NASH) | 935 |  |  |  |
| GSE  136103 | 10X  Genomics | [GSM4041150](https://www.ncbi.nlm.nih.gov/geo/query/acc.cgi?acc=GSM4041150) | Health | 1394 | Male | NA | |
|  |  | [GSM4041153](https://www.ncbi.nlm.nih.gov/geo/query/acc.cgi?acc=GSM4041153) | Health | 6435 | Male |  |  |
|  |  | [GSM4041155](https://www.ncbi.nlm.nih.gov/geo/query/acc.cgi?acc=GSM4041155) | Health | 2432 | Male |  |  |
|  |  | [GSM4041158](https://www.ncbi.nlm.nih.gov/geo/query/acc.cgi?acc=GSM4041158) | Health | 4040 | Female |  |  |
|  |  | [GSM4041160](https://www.ncbi.nlm.nih.gov/geo/query/acc.cgi?acc=GSM4041160) | Health | 4312 | Male |  |  |
|  |  | [GSM4041161](https://www.ncbi.nlm.nih.gov/geo/query/acc.cgi?acc=GSM4041161) | Cirrhosis (NAFLD-induced fibrotic) | 1563 | Female |  |  |
|  |  | [GSM4041164](https://www.ncbi.nlm.nih.gov/geo/query/acc.cgi?acc=GSM4041164) | Cirrhosis (Alcohol- induced fibrotic) | 2333 | Male |  |  |
|  |  | [GSM4041166](https://www.ncbi.nlm.nih.gov/geo/query/acc.cgi?acc=GSM4041166) | Cirrhosis (Alcohol- induced fibrotic) | 1019 | Male |  |  |
|  |  | [GSM4041168](https://www.ncbi.nlm.nih.gov/geo/query/acc.cgi?acc=GSM4041168) | Cirrhosis (NAFLD-induced fibrotic) | 4383 | Male |  |  |
|  |  | [GSM4041169](https://www.ncbi.nlm.nih.gov/geo/query/acc.cgi?acc=GSM4041169) | Cirrhosis (PBC-induced fibrotic) | 2245 | Female |  |  |
| GSE  125449 | 10X  Genomics | GSM4050085 | Cancer (HCC) | 48 | Male | 61 | White |
|  |  | [GSM4050086](https://www.ncbi.nlm.nih.gov/geo/query/acc.cgi?acc=GSM4050086) | Cancer (HCC) | 175 | Male | 77 | White |
|  |  | [GSM4050088](https://www.ncbi.nlm.nih.gov/geo/query/acc.cgi?acc=GSM4050088) | Cancer (ICC) | 12 | Female | 47 | White |
|  |  | [GSM4050089](https://www.ncbi.nlm.nih.gov/geo/query/acc.cgi?acc=GSM4050089) | Cancer (ICC) | 56 | Male | 63 | White |
|  |  | [GSM4050090](https://www.ncbi.nlm.nih.gov/geo/query/acc.cgi?acc=GSM4050090) | Cancer (HCC) | 68 | Female | 63 | Black |
|  |  | [GSM4050091](https://www.ncbi.nlm.nih.gov/geo/query/acc.cgi?acc=GSM4050091) | Cancer (ICC) | 305 | Male | 61 | White |
|  |  | [GSM4050092](https://www.ncbi.nlm.nih.gov/geo/query/acc.cgi?acc=GSM4050092) | Cancer (HCC) | 672 | Male | 63 | Black |
|  |  | [GSM4050093](https://www.ncbi.nlm.nih.gov/geo/query/acc.cgi?acc=GSM4050093) | Cancer (ICC) | 58 | Female | 64 | White |
|  |  | [GSM4050094](https://www.ncbi.nlm.nih.gov/geo/query/acc.cgi?acc=GSM4050094) | Cancer (HCC) | 29 | Male | 65 | Black |
|  |  | [GSM4050095](https://www.ncbi.nlm.nih.gov/geo/query/acc.cgi?acc=GSM4050095) | Cancer (HCC) | 128 | Male | 74 | Asian |
|  |  | [GSM4050096](https://www.ncbi.nlm.nih.gov/geo/query/acc.cgi?acc=GSM4050096) | Cancer (ICC) | 284 | Male | 61 | White |
|  |  | [GSM4050098](https://www.ncbi.nlm.nih.gov/geo/query/acc.cgi?acc=GSM4050098) | Cancer (HCC) | 85 | Male | 63 | White |
|  |  | [GSM4050100](https://www.ncbi.nlm.nih.gov/geo/query/acc.cgi?acc=GSM4050100) | Cancer (ICC) | 365 | Male | 67 | Asian |
|  |  | [GSM4050102](https://www.ncbi.nlm.nih.gov/geo/query/acc.cgi?acc=GSM4050102) | Cancer (ICC) | 45 | Male | 69 | White |
|  |  | [GSM4050104](https://www.ncbi.nlm.nih.gov/geo/query/acc.cgi?acc=GSM4050104) | Cancer (ICC) | 40 | Female | 52 | White |
|  |  | [GSM4050106](https://www.ncbi.nlm.nih.gov/geo/query/acc.cgi?acc=GSM4050106) | Cancer (ICC) | 1166 | Female | 80 | White |
|  |  | [GSM4050108](https://www.ncbi.nlm.nih.gov/geo/query/acc.cgi?acc=GSM4050108) | Cancer (HCC) | 66 | Female | 62 | White |
|  |  | [GSM4050110](https://www.ncbi.nlm.nih.gov/geo/query/acc.cgi?acc=GSM4050110) | Cancer (ICC) | 140 | Female | 71 | White |

Note: NASH=Non-alcoholic steatohepatitis, NA=Not applicable, NAFLD=Non-alcoholic fatty liver disease, PBC=Primary biliary cholangitis, HCC=Hepatocellular carcinoma, ICC=Intrahepatic cholangiocarcinoma.

**Table S2. The representative genes of M1 and M2 polarization.**

| M1 polarization | IL12, IL23, IL12, TNF, IL6, CD86, IL1B, MARCO, NOS2, IL12, CD64, CD80, CXCR10, IL23, CXCL9, CXCL10, CXCL11, CD86, IL1A, IL1B, IL6, CCL5, IRF5, IRF1, CD40, IDO1, KYNU, CCR7 |
| --- | --- |
| M2 polarization | ARG1, ARG2, IL10, CD32, CD163, CD23, CD200R1, PDCD1LG2, CD274, MARCO, CSF1R, CD206, IL1RN, IL1R2, IL4R, CCL4, CCL13, CCL20, CCL17, CCL18, CCL22, CCL24, LYVE1, VEGFA, VEGFB, VEGFC, VEGFD, EGF, CTSA, CTSB, CSTC, CTSD, TGFB1, TGFB2, TGFB3, MMP14, MMP19, MMP9, CLEC7A, WNT7B, FASL, TNFSF12, TNFSF8, CD276, VTCN1, MSR1, FN1, IRF4 |

**Table S3. The transcription factors of top 1 regulons from macrophage and naïve CD4+ T cells in cancer.**

| Macrophage (cancer) | AASDH, ATP5A1, ATP5I, CAPN1, CD63, CHCHD1, CHMP5, CHST2, DDIT4, FUCA1,  IFIT3, LRPAP1, MPP7, NUDC, PPP3CA, PSMB5, SLC19A2, SMPD1, SPON2, STK38L, TFDP2, VMAC, B4GALT4, NAALADL1, ZNF816, ANAPC2, ARL4C, BOD1, C1orf109, CBX6, CCPG1, CD2BP2, CLTC, DNAJB12, EHD4, ENPP4, FKBP11, GTF3C1, GZMB, KLF2, MAGOH, MPP5, NEU1, PPIB, RAB11FIP3, RHOC, RPL18, RPL39, RPS29, TBL3, TMEM30A, USO1, ZBTB16, ADNP, ARMCX3, BRD2, CDKN1A, DNAJC25, FAM91A1, HINT1, IVNS1ABP, KDELR2, LASP1, MAP3K10, MGA, RBM15B, RHEB, RPL36AL, RPN1, SGMS1, SGTA, SMAD3, STAG1, TBC1D15, TM9SF2, TMEM222, TOLLIP, ZFP36L1 |
| --- | --- |
| Naïve CD4+ T cell (cancer) | CALU, MLEC, MOSPD1, SEC61B, STT3A, TXNDC11, CHPF, COMMD6, BTF3, CD59, DNAJC1, EDEM1, JMY, NARF, NME1, NOL9, SLC43A1, SPPL3, UPRT, OSBP, RPS3, ZFP36L1, C6orf89, E2F5, RUVBL2, C16orf74, CREB3L2, FAM3C, FAM46C, HSPA13, MUT, PDDC1, PRR5, PTMA, RAB30, RPL17, RPL37A, RPS16, SEC61A1, CAPZB, CFL1, RPL7, RPS13, RPS24, TECR, XBP1, ZNF296 |
